# Global Invasion History and Genomic Signatures of Adaptation of the Highly Invasive Sycamore Lace Bug

**DOI:** 10.1093/gpbjnl/qzae074

**Published:** 2024-10-14

**Authors:** Zhenyong Du, Xuan Wang, Yuange Duan, Shanlin Liu, Li Tian, Fan Song, Wanzhi Cai, Hu Li

**Affiliations:** Department of Entomology and MOA Key Lab of Pest Monitoring and Green Management, College of Plant Protection, China Agricultural University, Beijing 100193, China; Department of Entomology and MOA Key Lab of Pest Monitoring and Green Management, College of Plant Protection, China Agricultural University, Beijing 100193, China; Department of Entomology and MOA Key Lab of Pest Monitoring and Green Management, College of Plant Protection, China Agricultural University, Beijing 100193, China; Department of Entomology and MOA Key Lab of Pest Monitoring and Green Management, College of Plant Protection, China Agricultural University, Beijing 100193, China; Department of Entomology and MOA Key Lab of Pest Monitoring and Green Management, College of Plant Protection, China Agricultural University, Beijing 100193, China; Department of Entomology and MOA Key Lab of Pest Monitoring and Green Management, College of Plant Protection, China Agricultural University, Beijing 100193, China; Department of Entomology and MOA Key Lab of Pest Monitoring and Green Management, College of Plant Protection, China Agricultural University, Beijing 100193, China; Department of Entomology and MOA Key Lab of Pest Monitoring and Green Management, College of Plant Protection, China Agricultural University, Beijing 100193, China

**Keywords:** *Corythucha ciliata*, Sycamore lace bug, Genomic architecture, Adaptation, Balancing selection

## Abstract

Invasive species cause massive economic and ecological damages. Climate change has resulted in an unprecedented increase in the number and impact of invasive species; however, the mechanisms underlying these invasions are unclear. The sycamore lace bug, *Corythucha ciliata*, is a highly invasive species originating from North America and has expanded across the Northern Hemisphere since the 1960s. In this study, we assembled the *C. ciliata* genome using high-coverage Pacific Biosciences (PacBio), Illumina, and high-throughput chromosome conformation capture (Hi-C) sequencing. A total of 15,278 protein-coding genes were identified, and expansions of gene families with oxidoreductase and metabolic activities were observed. In-depth resequencing of 402 samples from native and nine invaded countries across three continents revealed 2.74 million single nucleotide polymorphisms. Two major invasion routes of *C. ciliata* were identified from North America to Europe and Japan, with a contact zone forming in East Asia. Genomic signatures of selection associated with invasion and long-term balancing selection in native ranges were identified. These genomic signatures overlapped with each other as well as with expanded genes, suggesting improvements in the oxidative stress and thermal tolerance of *C. ciliata*. These findings offer valuable insights into the genomic architecture and adaptive evolution underlying the invasive capabilities of species during rapid environmental changes.

## Introduction

Biological invasions threaten ecosystems, biodiversity, public health, and welfare, leading to enormous economic losses [1,2]. Global climate change, particularly rising temperatures, has resulted in an unprecedented increase in the number and impact of invasive species, with invasive ranges often exceeding native ranges [3,4]. As only a small proportion of introduced species become effective invaders, elucidating the mechanisms underlying successful invasions is essential [5,6]. Various hypotheses, including those related to transport opportunities, propagule pressure, habitat matching, fecundity, and population size, have been proposed [7,8]; however, consistent empirical evidence across different invasion scenarios and organisms is lacking. Furthermore, few studies have investigated the genomic signatures that may underlie these variations.

Pre- and post-introduction adaptations contribute to successful invasion and establishment. The environmental differences between introduced and native ranges can impose strong selective pressures, necessitating genetic adaptation to novel habitats for colonization and invasion [8,9]. Natural selection frequently favors only a few fitness-enhancing genes [10,11], and evolutionary rates can be high during invasion [12,13]. In addition, an invasive species may be pre-adapted to some or most aspects of the new environments. Phenotypic plasticity alone may be sufficient for invaders to survive and establish in invasive ranges, particularly if the native and invasive environments are highly similar [8]. The potential for pre-introduction invasive capacity may be influenced by the genomic architecture of the species [8,9,14], along with the breadth of their native distribution in fluctuating environments, favoring a wide array of alleles at a given locus to maintain functional polymorphisms [5,8,13,15,16].

The sycamore lace bug, *Corythucha ciliata* (Hemiptera: Tingidae), is an oligophagous grazer specialized to sycamore trees (*Platanus* spp.). Both *C. ciliata* nymphs and adults feed on the underside of leaves, resulting in a white stippling [17], which can lead to chlorotic or bronzed leaf foliage, tree growth inhibition, premature senescence, and even tree death. Thus, the widespread invasion of sycamore lace bug populations has severely impacted urban afforestation because of the popularity of sycamore trees as street trees worldwide. The sycamore lace bug is native to North America [17,18]. It was first discovered outside its native ranges in Padova, Italy, in 1964 and has since been recorded in various southern and central European countries. Between 1995 and 2002, *C. ciliata* also invaded South Korea, China, and Japan [19]. Our sampling from 2016 to 2019 (Table S1) revealed that its distribution range in East Asia has spread to most cities in southwestern, northeastern, eastern, and central China, as well as to most cities in South Korea and the Honshu, Shikoku, and Kyushu regions in Japan. Additionally, the lace bug has been discovered in Chile, Australia, and South Africa. Despite rising temperatures due to climate change, this temperate species has also successfully invaded multiple subtropical regions [19]. However, the global invasion routes and the mechanisms underlying the rapid invasion and variation between invasive and native ranges remain unclear.

Successful invasion often depends on pre- and post-introduction adaptations; however, the mechanisms driving this success are unclear. We hypothesize that certain genomic features, particularly the expansion of specific gene families and selection for pivotal traits, may be crucial to invasiveness. To test this hypothesis, *C. ciliata* was used as a target invasive species in this study. We aimed to (1) identify species-specific gene families that have undergone expansion across the genome; (2) decipher genomic signatures of selection during various invasion scenarios; (3) investigate signatures of selection within native habitats; and (4) assess the interplay between these adaptive genomic features. By addressing these objectives, we aim to elucidate the genomic architecture that facilitates the invasive prowess of species like *C. ciliata*.

## Results

### Chromosome-level genome assembly of *C. ciliata*

**Figure 1:**
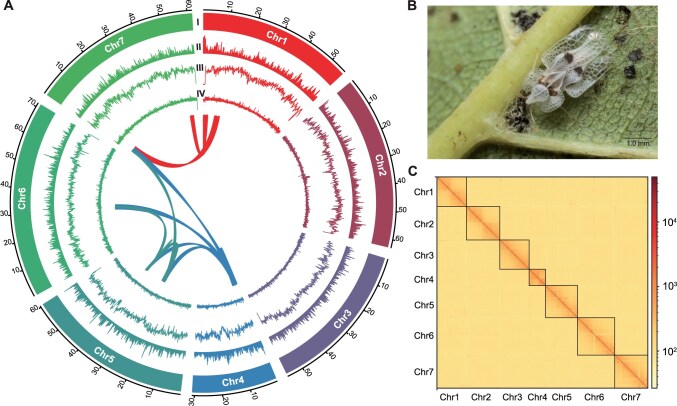
Genome architecture of *C. ciliata* **A**. Circos plot exhibiting the landscape of the *C. ciliata* genome. (I) Seven chromosomes on the Mb scale. (II) Density of protein-coding genes. (III) Distribution of repeat sequences. (IV) Distribution of GC content. The distributions of protein-coding genes and repeat sequences are calculated in a 100-kb non-overlapping sliding window. The distribution of GC content is calculated in a 10-kb window. The ribbon inside the circle represents the synteny block within the genome. **B**. *C. ciliata* feeding on a sycamore leaf. **C**. Hi-C contact map of seven chromosomes. Chr, chromosome; Hi-C, high-throughput chromosome conformation capture.

Pacific Biosciences (PacBio) long-read sequencing and assembly, Illumina short-read polishing, and high-throughput chromosome conformation capture (Hi-C) scaffolding were used to assemble the *C. ciliata* genome (**Figure 1**A). Using high-molecular-weight (HMW) DNA extracted from *C. ciliata* samples (Figure 1B), 9,158,256 PacBio reads were obtained, corresponding to 120.03-Gb data and 302× genome coverage. The *k*-mer analysis estimated the haploid genome size at ∼ 400 Mb (Figure S1). The initial assembly resulted in 1085 contigs, with a total length of 400.78 Mb and a contig N50 of 1.19 Mb. This assembly was further scaffolded based on the 61.62-Gb Hi-C data (∼ 154×), resulting in a 401.27-Mb final assembly with a scaffold N50 of 60.52 Mb. We anchored 99.67% of the assembly, equivalent to 399.93 Mb, onto seven chromosomes, consistent with findings from previous karyotype studies [20] (Figure 1A and C; Table S2). Benchmarking Universal Single-Copy Orthologs (BUSCO) analysis indicated that the *C. ciliata* genome captured 95.3% (93.7% single-copy and 1.6% duplicated) of complete BUSCOs (1579 out of 1658 in Insecta odb10).

### Comparative genomic analyses between *C. ciliata* and other hemipterans

**Figure 2:**
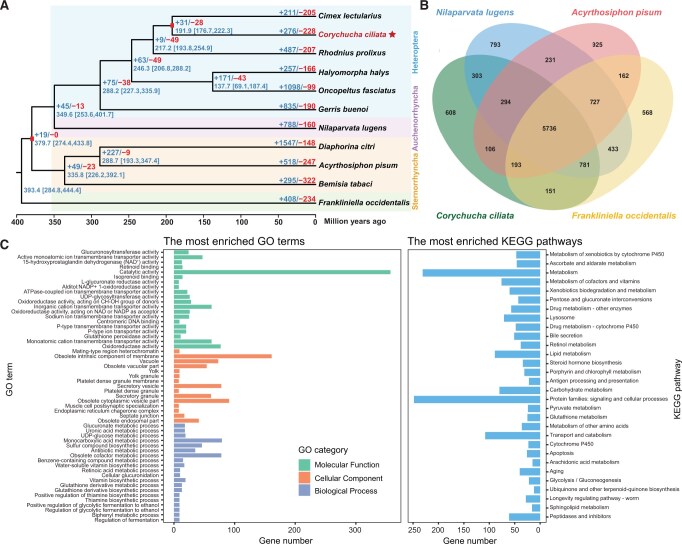
Comparative genomic analyses of hemipteran insects **A**. Phylogeny, divergence time, and gene family expansion and contraction of ten hemipterans with the thrips *Frankliniella occidentalis* as an outgroup. The phylogenetic tree was reconstructed using RAxML. All nodes show 100% bootstrap support. Divergence time was estimated using MCMCTree, with two calibrated nodes marked with red rectangles. The mean divergence time is shown at each node, with the bracket indicating the 95% highest posterior densities. The numbers of expanded (blue) and contracted (red) gene families are indicated on the branch next to each node. **B**. Venn diagram showing the numbers of gene families shared among four paraneopteran species. **C**. GO and KEGG enrichment analyses of the expanded genes in the *C. ciliata* genome. GO terms and KEGG pathways are sorted according to the *P* value (higher *P* values toward the bottom). Only the top 20 GO terms of the Biological Process and Molecular Function categories, the top 15 GO terms of the Cellular Component category, and the top 30 KEGG pathways are shown. The complete list of enriched GO terms and KEGG pathways is provided in Tables S5 and S6. GO, Gene Ontology; KEGG, Kyoto Encyclopedia of Genes and Genomes.

A total of 15,278 protein-coding genes were identified in the *C. ciliata* genome based on the combination of full-length transcriptome-based prediction, homology-based prediction, and *ab initio* prediction, covering 206.29 Mb in length (Table S3). Of these genes, 13,235 (86.63%) were functionally annotated using five databases (Figures S2 and S3). For comparative analyses, orthologous groups (also known as gene families) were identified across ten hemipteran insects, using the thrips *Frankliniella occidentalis* as an outgroup. A phylogenetic tree was reconstructed using a matrix of 895 concatenated single-copy orthologous genes that supported the phylogenetic framework of Hemiptera ((Auchenorrhyncha + Heteroptera) + Sternorrhyncha) (**Figure 2**A). A substantial number (5736) of shared gene families among four Hemiptera and Paraneoptera species were identified (Figure 2B).

Gene family expansion and contraction analysis was performed across the hemipteran (paraneopteran) phylogeny to examine the genomic mechanism underlying the adaptive capacity of *C. ciliata*. A total of 276 expanded gene families corresponding to 1171 genes and 228 contracted gene families corresponding to 402 genes were identified in the *C. ciliata* genome (Table S4). Gene Ontology (GO) and Kyoto Encyclopedia of Genes and Genomes (KEGG) enrichment analyses were performed to identify the potential functions of these genes. The expanded genes were significantly enriched for oxidoreductase and metabolism-related functions. In the Molecular Function (MF) category, the most significantly enriched GO terms included “15-hydroxyprostaglandin dehydrogenase (NAD^+^) activity” (GO:0016404), “L-glucuronate reductase activity” (GO:0047939), “oxidoreductase activity” (GO:0016491), “alditol:NADP^+^ 1-oxidoreductase activity” (GO:0004032), and “glutathione peroxidase activity” (GO:0004602) (Figure 2C; Table S5). The most significantly enriched Biological Process (BP) GO terms were “glucuronate metabolic process” (GO:0019585), “uronic acid metabolic process” (GO:0006063), and “UDP-glucose metabolic process” (GO:0006011) (Figure 2C; Table S5). The “catalytic activity” (GO:0003824) and “obsolete intrinsic component of membrane” (GO:0031224) were the GO terms with the most enriched genes. Among the KEGG pathways, “metabolism of xenobiotics by cytochrome P450”, “ascorbate and aldarate metabolism”, and “metabolism” were the most significantly enriched pathways. Overall, the categories of “metabolism”, “protein families: signaling and cellular processes”, and “transport and catabolism” had the greatest number of enriched pathways (Figure 2C; Table S6). In comparison, the contracted genes were significantly enriched for sterol, cholesterol, and lipid transfer functions, as well as for development and morphogenesis. The most significantly enriched GO terms included “ABC-type sterol transporter activity” (GO:0034041), “cholesterol transfer activity” (GO:0120020), and “lipid transfer activity” (GO:0120013) in the MP category, and “ecdysteroid metabolic process” (GO:0045455) and “apoptotic process involved in morphogenesis” (GO:0060561) in the BP category (Tables S7 and S8).

### Large-scale genome resequencing and genomic variations in *C. ciliata*

**Figure 3:**
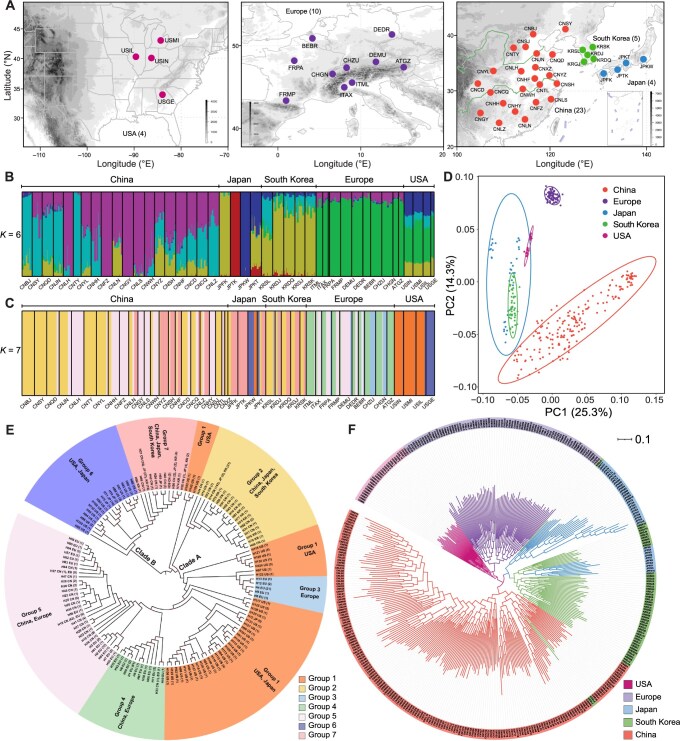
Population sampling and genetic structure of *C. ciliata* based on genomic SNPs and mitogenomes **A**. Sample locations in North America, Europe, and East Asia. Different colors indicate sample locations in five different regions, *i.e.*, the USA (amaranth), Europe (purple), Japan (blue), South Korea (green), and China (orange). The base map is officially approved with Approval No. GS(2019)1682 (http://bzdt.ch.mnr.gov.cn/). **B**. Genetic structure revealed by ADMIXTURE based on the genomic SNPs in 370 samples. Each color represents one of the six ancestral genomic components, and each vertical bar represents an individual. **C**. Genetic structure revealed by BAPS based on the mitogenomes of 665 samples. Each color represents one of the seven BAPS groups, and each vertical bar represents an individual. **D**. PCA plot based on the genomic SNPs of 370 samples, with PC1 plotted against PC2. **E**. Phylogenetic tree reconstructed by IQ-tree based on mitogenomic haplotypes. Table S23 presents individual information on haplotypes. Different colors represent seven different BAPS groups. Different regions are shown on the tree diagram. Red circles at the nodes represent bootstrap values ≥ 70. **F**. Phylogenetic tree reconstructed by RAxML based on genomic SNPs in 370 samples. Different colors represent five different regions. All nodes have a bootstrap value of 100 except for those with gray circles. BAPS, Bayesian Analysis of Population Structure; PCA, principal component analysis; SNP, single nucleotide polymorphism.

In total, 410 *C. ciliata* samples from native and nine invaded countries across three continents were sequenced (**Figure 3**A). Eight of these samples were removed owing to low mapping rates (Table S9); however, four of them were used for mitochondrial genome (mitogenome) assembly. The remaining 402 samples were used for single nucleotide polymorphism (SNP) genotyping, with an average coverage depth of 22.12× (ranging from 9.03× to 42.9×) (Table S9). Mapping rates to the reference genome ranged from 66.61% to 97.68%, with an average of 95.84% (Table S9). Thirteen samples were excluded because of high missing rates in the SNP dataset (Table S10). Nineteen individuals were identified as full-siblings of another individual within the population and excluded based on the kinship coefficient (Table S11). A total of 2.74 million SNPs from 370 samples were further analyzed.

The SNPs were filtered and pruned to maintain a single SNP per 3-kb window using PLINK to account for linkage disequilibrium (LD) and to avoid bias in the population genetic structure, resulting in 106,761 independent loci that were used to analyze population genetic structure.

A mitochondrial dataset comprising 665 whole mitogenomes was assembled to resolve the evolutionary history of *C. ciliata* and leverage the advantages of the haploid organelle genome in reflecting the recent divergence history [21–23]. This dataset included 406 samples sequenced in the present study and 259 obtained from our previous skim sequencing data [22,23] (Table S1). This dataset represents a robust resource for resolving the global invasion history and adaptation of *C. ciliata*.

### Global population genetic structure and migration pattern of *C. ciliata*

**Figure 4:**
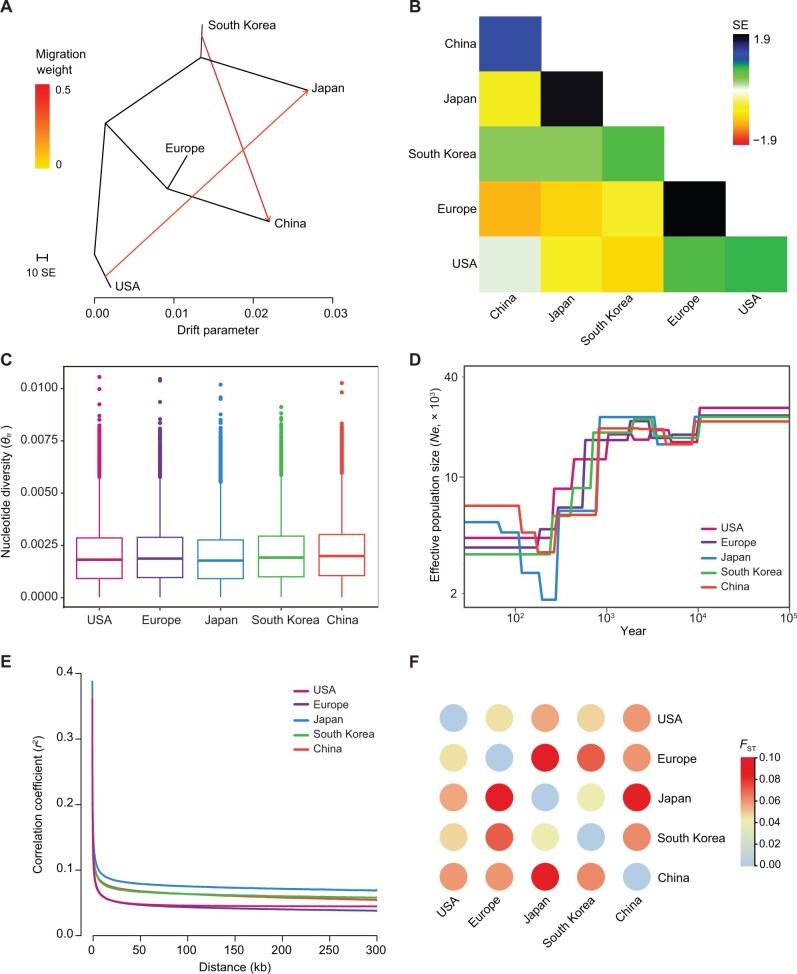
Migration, genetic diversity, demographic history, and genetic differentiation of *C. ciliata* **A**. TreeMix phylogeny with two migration events modeled. The horizontal branch lengths are proportional to the amount of genetic drift that has occurred along that branch. The scale bar shows 10 times the average SE of the entries in the sample covariance matrix. **B**. Residual fit of the observed *vs.* the predicted squared allele frequency differences, expressed as the number of SE of the deviation. Residuals above zero represent sites that are more closely related to each other in the data than in the tree. **C**. Genome-wide mean nucleotide diversity (*θ*_π_) under a 3-kb sliding window. **D**. Demographic history, depicted by the changes in effective population size (*Ne*) over time and inferred by SMC++. **E**. LD decay between SNPs as measured using the coefficient of correlation (*r*^2^) of alleles at any two loci. **F**. Heatmap of Weir and Cockerham’s mean estimate of *F*_ST_ among five different countries and regions. SE, standard error.

Population genetic structure analyses (Figure 3B and C), principal component analysis (PCA; Figure 3D), phylogenetic analyses (Figure 3E and F), and migration pattern analyses (**Figure 4**A and B) were conducted to reconstruct the global invasion routes of *C. ciliata*. ADMIXTURE analysis revealed that all populations exhibited six ancestry components (*K* = 6) based on the lowest error of cross-validation among *K* = 2–10 (Figure 3B, Figure S4). Native American populations harbored four major components, whereas European populations inherited only one of these four components. Populations in Japan, South Korea, and China showed high levels of admixture, with Chinese populations sharing components with European, South Korean, and Japanese populations. Specifically, the Japanese Honshu population exhibited three components, whereas the Shikoku and Kyushu populations had one. South Korean and Chinese populations shared two components (Figure 3B). The PCA and phylogenetic analyses supported the general clustering of samples from different regions, except for the Kyushu populations in Japan, which clustered with the South Korean samples (Figure 3D and F, Figure S5). Three South Korean samples were clustered with the Japanese Honshu and Chinese samples in the phylogenetic tree (Figure 3F). Furthermore, ADMIXTURE and PCA analyses within the five distinct regions revealed no admixture in American, South Korean, and European populations (*K* = 1). However, two ancestry components were detected in Chinese and Japanese populations (*K* = 2; Figure S6A and B), with a high degree of admixture noted in Chinese populations (Figures S6A and S7A), consistent with the broader analysis (Figure 3B).

The absence of recombination and the maternal inheritance patterns of the mitogenome led to divergence in genomic structure and phylogenetic relationships between the nuclear and mitochondrial datasets; however, the overall patterns remained similar. Bayesian Analysis of Population Structure (BAPS) resolved seven mitogenomic groups in the mitogenomic dataset (*K* = 7; Figure 3C; Table S12), corresponding to multiple lineages in two major clades of the phylogenetic tree (Figure 3E). In Clade A, Group 2 consisted of East Asian haplotypes, Group 3 consisted of European haplotypes, and Groups 4 and 5 consisted of Chinese and European Group 1-derived haplotypes, which included the American and Japanese Honshu haplotypes and was split into three lineages (Figure 3C and E). In Clade B, Group 7 comprised East Asian Group 6-derived haplotypes, including the American and Japanese Honshu haplotypes (Figure 3C and E).

TreeMix analysis based on genomic SNPs resolved two major clades diverged from American populations, containing the South Korean and Japanese populations, and European and Chinese populations, respectively (Figure 4A). Two migration events with high weight were identified, one from American to Japanese populations and another from South Korean to Chinese populations (Figure 4A). The residual plot indicated potential gene flow among American and European populations as well as among Japanese and South Korean populations (Figure 4B).

### Global genetic diversity and demographic history of *C. ciliata*

The genome-wide nucleotide diversity (*θ*_π_), calculated on sliding-windows, was highest in South Korean and Chinese populations (mean *θ*_π_ = 0.0021), whereas the Japanese populations had the lowest diversity (mean *θ*_π_ = 0.0019) (Figure 4C). When analyzing each sampling population based on all genomic SNPs, the highest observed heterozygosity (Ho) was observed in Chongqing, China, whereas the lowest was in Tokushima, Japan (Table S13). South Korean populations had the highest diversity (mean Ho = 0.27), and Japanese populations had the lowest diversity (mean Ho = 0.21). Notably, the highest Ho was observed in Georgia, USA, when analyzing these populations based on only variant SNPs, whereas the lowest Ho was in Qingdao, China (Table S13). The American populations had the highest diversity (mean Ho = 0.36), followed by the Japanese populations (mean Ho = 0.35), whereas the Chinese and South Korean populations had the lowest diversity (mean Ho = 0.33; Table S13). For the mitogenomic dataset, the highest diversity was identified in South Korean (π = 0.00333) and American populations (π = 0.00323), whereas the European population had the lowest diversity (π = 0.00074; Table S1). These results indicate that Chinese and South Korean populations possess higher diversity on fixed SNPs than the other populations, supporting the hypothesis that multiple introductions may have increased the genetic diversity in these populations.

The demographic history of *C. ciliata* was determined using SMC++ and Stairway Plot 2. The results suggested that the effective population size (*Ne*) of *C. ciliata* began to rapidly decrease 1000 years ago, reaching its lowest level in the last 200–300 years (Figure 4D, Figure S8). The Japanese populations showed the longest LD decay distance, whereas the American and European populations showed the shortest LD decay distances (Figure 4E).

Weir and Cockerham’s mean estimate of *F*_ST_ was generally lower for American populations (*F*_ST_ = 0.045–0.059) than that for the other regions (*F*_ST_ = 0.060–0.085), except for South Korea and Japan (*F*_ST_ = 0.041), which had the lowest *F*_ST_. Chinese populations had relatively low *F*_ST_ with populations from Europe (*F*_ST_ = 0.060) and South Korea (*F*_ST_ = 0.062) but had high *F*_ST_ with Japanese populations (*F*_ST_ = 0.084). The highest *F*_ST_ was observed between European and Japanese populations (*F*_ST_ = 0.085) (Figure 4F; Table S14). For the mitogenomic dataset, the highest pairwise *F*_ST_ was also observed between European and Japanese populations (*F*_ST_ = 0.729), followed by that between Chinese and Japanese populations (*F*_ST_ = 0.516). In contrast, the lowest *F*_ST_ was observed between American and South Korean populations (*F*_ST_ = 0.109), followed by that between American and Chinese populations (*F*_ST_ = 0.135) (Table S14).

### Genomic signatures of selection associated with invasion of *C. ciliata*

**Figure 5:**
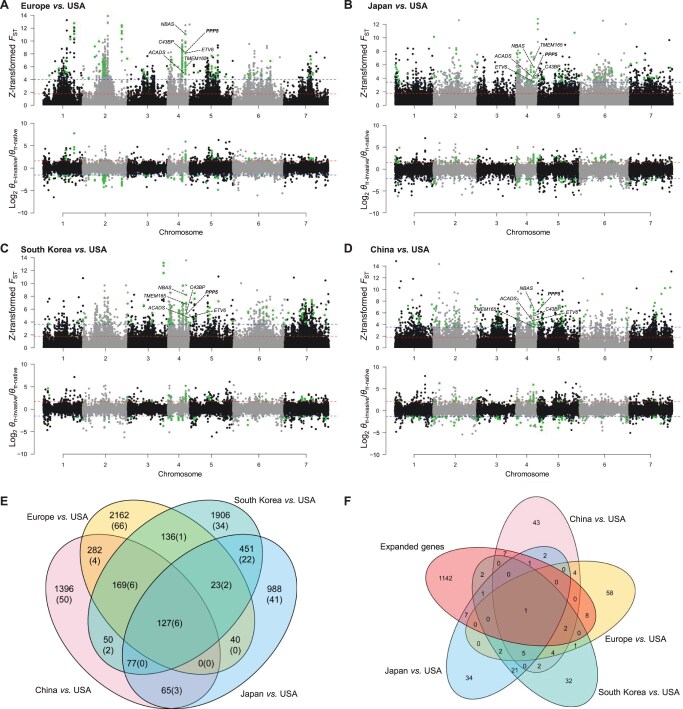
Genomic signatures of selection associated with invasion of *C. ciliata* **A**.–**D**. The upper Manhattan plots show selection signatures detected by *F*_ST_. The x-axis represents seven chromosomes of the *C. ciliata* genome, and the y-axis represents the values of *Z*-transformed *F*_ST_. The horizontal dashed lines represent the thresholds of top 1% (blue) and top 5% (red) values, respectively. The bottom plots show selection signatures detected by nucleotide diversity, *θ*_π_ ratio (*θ*_π-invasive_/*θ*_π-native_). The y-axis represents log_2_  *θ*_π-invasive_/*θ*_π-native_. The horizontal dashed lines represent the thresholds of the top 0.5% (red) and bottom 0.5% (blue) values, respectively. The highlighted dots represent the shared loci detected by two methods. **E**. Venn diagram showing the overlapping SNPs (genes) between those identified in four pairs of comparison between invasive and native populations. **F**. Venn diagram showing the overlapping genes between those identified in four pairs of comparison between invasive and native populations and expanded genes identified in comparative genomic analyses.

The genomes of all four invasive populations (Europe, Japan, South Korea, and China), as well as the native populations (USA), were scanned to determine whether selection has contributed to the successful invasion of *C. ciliata*. Because the direction of selection was unknown, both *F*_ST_ and *θ*_π_ ratio were applied to detect the genomic signatures of selection associated with invasion. A threshold of the top 1% values was used for *F*_ST_, and both the top and bottom 0.5% candidates were preserved for the *θ*_π_ ratio (**Figure 5**A–D). Only genomic loci shared by both methods were considered as selection targets to avoid false-positive signatures and the effects of population structure. As a result, 7872 SNPs were identified as genomic signatures of selection (Table S15), which were assigned to 237 genes (Table S16). Of these signatures, 18% (1420) of SNPs and 19.4% (46) of genes were shared between at least two comparisons of invasive and native populations (Figure 5E).

To investigate the potential effects of these signatures on the environmental adaptation of *C. ciliata*, environmental association analysis (EAA) was performed based on the 7872 SNPs and 10 bioclimatic variables which were selected after filtering out strongly corelated variables. With the criterion of log_10_ Bayes factor (BF) > 1.5 [24,25], allele frequency variations were found in ∼ 4% (294) SNPs and correlated with environmental variation across 42 sampling populations (Table S17). Of these SNPs, 35.4% (104) were associated with the maximum temperature of the warmest month (BIO5), followed by 15.0% (44) correlated with the mean temperature of the wettest quarter (BIO8) and 11.6% (34) correlated with the minimum temperature of the coldest month (BIO6), indicating their potential roles in temperature adaption of *C. ciliata*.

Notably, 127 shared SNPs on 6 genes were clustered on chromosome 4 over a ∼ 47-kb genomic region. These genes included neuroblastoma-amplified sequence (*NBAS*), ETS variant transcription factor 6 (*ETV6*), transmembrane protein 165 (*TMEM165*), collagen type IV alpha-3-binding protein (*C43BP*), serine/threonine-protein phosphatase 5 (*PPP5*), and short-chain specific acyl-CoA dehydrogenase (*ACADS*) (Figure 5A–E; Table S18). Higher nucleotide diversity was observed in this genomic region in invasive populations than in native populations, indicating that they may be favored by diversifying selection (Figure 5A–D). The same analysis was performed using 20 randomly selected samples from each region to exclude bias owing to sample size, and similar results were obtained (Figure S9; Table S19). However, one adjacent gene, chitooligosaccharidolytic β-*N*-acetylglucosaminidase (*BmChiNAG*), was included as a shared selection target in the cluster. Furthermore, *PPP5* was also included in the expanded gene set of the *C. ciliata* genome (Figure 5F).

### Widespread balancing selection signatures in native ranges of *C. ciliata*

To determine the origin of adaptation, we investigated whether the genomic signatures related to selection during invasion or the expanded genes of this species overlapped with the signatures of long-term balancing selection in native populations. The genomes of the native North American populations were scanned for signatures of long-term balancing selection using *β* scores and chromosomal SNPs with minor allele frequency (MAF) ≥ 0.15 [13]. High *β* scores in this analysis indicated an excessive number of SNPs at similar frequencies.

**Figure 6:**
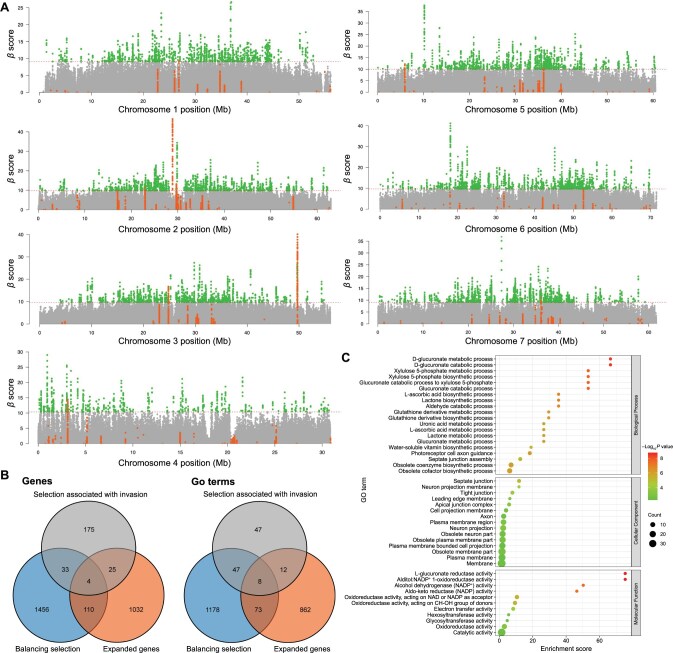
Genomic signatures of long-term balancing selection of *C. ciliata* in native ranges **A**. Manhattan plots showing balancing selection signatures in native populations detected by *β* scores on seven chromosomes of the *C. ciliata* genome. The horizontal dashed lines represent the threshold of the top 1% *β* scores. Green dots show SNPs that have top 1% *β* scores. Orange dots show candidate SNPs under selection and associated with invasion identified in four pairs of comparison between invasive and native populations. **B**. Venn diagrams showing the overlapping genes (left) and GO terms (right) between those identified in balancing selection, selection associated with invasion, and expanded genes. **C**. GO enrichment analysis of shared genes between balancing selection and expanded gene set.

Notably, the top 1% of candidate SNPs with signatures of long-term balancing selection were widespread across the seven chromosomes (**Figure 6**A). Whether the genomic loci associated with invasion exhibited strong signatures of long-term balancing selection compared to the whole genome in the native populations was investigated (Table S20). The candidate SNPs demonstrated significantly higher *β* scores than other genomic regions across all seven chromosomes (mean *β* = 3.13 *vs.* mean *β* = 1.58; Mann–Whitney *U* test, *U* = 3.8E−09, *P* = 5.3E−51). When analyzing the chromosomes separately, candidate SNPs identified on chromosome 2 (mean *β* = 4.56 *vs.* mean *β* = 1.61; Mann–Whitney *U* test, *U* = 1.9E−08, *P* = 4.1E−84) and chromosome 3 (mean *β* = 7.19 *vs.* mean *β* = 1.59; Mann–Whitney *U* test, *U* = 1.0E−08, *P* = 6.4E−54) had significantly higher *β* scores than other chromosomal regions. Interestingly, 33 sites were identified on chromosome 2 and 40 sites on chromosome 3 with the highest *β* scores, all identified as the candidate SNPs of selection associated with invasion (Figure 6A).

The top 1% of candidate SNPs with signatures of long-term balancing selection corresponded to 1603 genes (Table S21), which overlapped with 15.6% of the genes with selection signatures associated with invasion and 9.7% of the expanded genes (Figure 6B). The expanded genes also overlapped with 12.2% of the genes with selection signatures associated with invasion (Figure 6B). The overlap between the significantly enriched GO terms in these gene sets was investigated to determine the potential functions of these genes. In total, 41.2% of GO terms in the gene set with selection signatures associated with invasion and 8.5% of GO terms in the expanded gene set were shared with the GO terms in the gene set with balancing selection signatures (Figure 6B). The genes shared between genes with balancing selection signatures and expanded genes were enriched in oxidoreductase and metabolic activities (Figure 6C; Table S22), consistent with the GO enrichment results of the expanded genes (Figure 2C). For instance, “L-glucuronate reductase activity” (GO:0047939), “alditol:NADP^+^ 1-oxidoreductase activity” (GO:0004032), “oxidoreductase activity, acting on the CH-OH group of donors, NAD or NADP as acceptor” (GO:0016616), “oxidoreductase activity, acting on CH-OH group of donors” (GO:0016614), “oxidoreductase activity” (GO:0016491), and “catalytic activity” (GO:0003824) were the top GO terms enriched for the expanded genes of the *C. ciliata* genome (Figures 2C and 6C).

## Discussion

With ∼ 2000 described species, lace bugs are distributed worldwide, are usually host-specific, and can be highly destructive to plants. Understanding the mechanisms underlying invasiveness is essential to elucidate the evolutionary origins of adaptive traits that facilitate successful invasions. In the present study, we assembled a complete and highly contiguous genome of *C. ciliata*, excluding the Y chromosome, which is the first assembly and chromosome-level reference genome of Tingidae. The novel reference genome assembled in this study filled a knowledge gap in the genomics of lace bugs and may provide a crucial genomic reference for future research on this ecologically and economically essential group of insects.

### Gene family evolution in the *C. ciliata* genome

Based on comparative genomic analyses of ten hemipteran insects, we observed 276 expanded gene families associated with oxidoreductase and metabolism-related functions (Figure 2A and C; Tables S5 and S6). This finding contrasts with observations in many polyphagous insects, whose invasiveness is attributed to an increased capacity to use new nutritional sources. For instance, the gustatory receptor genes in the fall webworm [9] and the olfactory receptor genes in the codling moth [14] have expanded to facilitate their successful and rapid adaptation. *C. ciliata* was unlikely to use new nutrition sources or host plants to enable fast and successful establishment; instead, the decisive factor in their distribution and invasion appears to be their capacity to adapt to a wide temperature range [26]. Previous physiological studies reported that high temperatures induced oxidative stress, leading to high anaerobic respiration and antioxidant defenses in *C. ciliata* in the laboratory and field. This mechanism protects against oxidative damage caused by reactive oxygen species accumulation [27]. Strong enzymatic and metabolic activities, particularly the oxidoreductase activity, may provide high tolerance to oxidative stress under varying temperatures.

In the *C. ciliata* genome, 228 contracted gene families were identified (Figure 2A), which were significantly enriched for the ATP-binding cassette (ABC) transporters, mediators of the transport of a variety of physiologic lipid compounds (sterol, cholesterol, and other lipids), as well as for development and morphogenesis (Tables S7 and S8). The ABC transporter and cytochrome P450 gene families are typically associated with pesticide and xenobiotic resistance in insects [14,28,29]. However, the cytochrome P450 gene family was significantly expanded in the *C. ciliata* genome, while the ABC transporter gene family exhibited significant contraction. This specific contraction of the ABC transporter gene family may be related to the oligophagy of *C. ciliata* and its prolonged co-evolution with its host, sycamore trees. Such evolutionary dynamics suggest that while certain resistances are necessary and thus maintained, other redundant resistances may be lost over time.

### Global invasion history of *C. ciliata*

Two major invasion routes of *C. ciliata* were identified based on a vast population genomic dataset with 2.74 million SNPs and a comprehensive sampling covering native and invasive ranges across three continents. During an invasion, a strong bottleneck and inbreeding may reduce the genetic diversity of founder populations [30,31]. The lowest genome-wide nucleotide diversity was observed in Japanese populations, while the lowest mitogenomic diversity was observed in European populations; these findings are consistent with these effects (Figure 4C; Table S1). The most pronounced genetic differentiation was observed between European and Japanese populations (Figure 4F; Table S14), and the genetic structure and migration analyses (Figures 3B–F, 4A and B) supported that the European and Japanese Honshu populations were independently derived from two distinct invasion events. The Chinese and South Korean populations were derived from both invasion routes, indicating the existence of a contact zone in East Asia.

Interestingly, although the native American populations possessed the highest genetic diversity of variant sites (Table S13), the Chinese and South Korean populations exhibited higher nucleotide diversity than the native American and earlier introduced European populations [17,18] (Figure 4C; Table S1). This result was possibly caused by the genetic admixture of different invasive sources; nevertheless, the larger LD decay distance observed in the Chinese and South Korean populations was consistent with their more recent invasion history (Figure 4E). Multiple introductions have ameliorated the loss of genetic diversity during invasion and may facilitate the fitness of invasive populations [32,33].

Understanding the impact of anthropological activities on biological invasion and population dynamics can inform strategies for managing invasive species and conserving biodiversity. Given the limited flight capacity of *C. ciliata*, long-distance dispersion, particularly across continents, was possibly accomplished through anthropogenic activities. Urban afforestation facilitated international and domestic transportation of sycamore trees, providing opportunities for this insect to spread across geographic barriers [19]. East Asia, particularly regions of eastern China, South Korea, and Japan, are invasion hotspots [22]. Furthermore, a drastic population decrease was observed from 1000 to 200 years ago, which may have been associated with the exploitation and urbanization of North America, along with the possible logging of sycamore trees, a key source of wood at the time.

### Natural selection during the global invasion of *C. ciliata*

Environmental differences between native and invasive ranges may result in divergent selective pressures and genomic changes in crucial loci in novel habitats, which may enhance the fitness and invasiveness of invasive populations [8,9]. In this study, we examined the hypothesis that selection may improve the thermal adaptation of populations in invasive ranges and lead to varying success compared to native populations. Invasive species populations are sometimes more successful than native populations owing to a higher thermal tolerance as global climate change drives the increase in temperatures [3]. Based on the selection signature and environmental association analyses conducted in the study, a number of candidate SNPs (∼ 4%) under selection and associated with invasion were correlated with environmental variables, especially the highest and lowest temperature across their distribution ranges (Table S17). These results supported the hypothesis that selection may enhance the temperature adaptation of invasive populations, contributing to their higher thermal tolerance and rapid invasion.

Notably, *PPP5* and adjacent gene blocks were repeatedly selected during multiple invasion scenarios (Figure 5A–D). *PPP5* is involved in various cellular signaling pathways in higher eukaryotes, including those initiated by atrial natriuretic peptides, oxidative stress, glucocorticoids, and pathways operating through G-proteins [34,35]. Additionally, PPP5 binds to heat shock proteins (HSPs), specifically HSP70 and HSP90, through its regulatory tetratricopeptide repeat domain. This interaction enhances the stress tolerance of *C. ciliata* and contributes to the invasive success of many other species [3,35–37]. *PPP5* and *ETV6*, both within this gene block, are involved in the mitogen-activated protein kinase (MAPK) signaling pathway in the KEGG database. MAPK signaling cascades convey signals intracellularly via sequential protein phosphorylation and contribute to oxidative stress tolerance and metabolism [38]. This close genetic linkage may be favored by selection with reduced recombination [39,40]. This gene block was repeatedly selected during independent *C. ciliata* invasions and may play a key role in increasing their thermal tolerance in invasive ranges, resulting in varying success. Despite the rise in temperature attributed to climate change, this may also have facilitated the extensive invasion of numerous subtropical regions by this temperate species [19].

### Balancing selection in the native ranges of *C. ciliata*

Fluctuating environments may induce balancing selection, favoring multiple alleles at a given locus to retain functional polymorphisms [8,13]. *C. ciliata* spans a wide latitudinal range and has multiple overlapping generations annually. These bugs overwinter as adults under crevices, leaf litter, and loose bark [17,41]. Thus, *C. ciliata* frequently experiences temperature fluctuation, including hot summers (36°C to 40°C) and cold overwintering (–30°C to –10°C) [42,43]. Balancing selection is likely the major force maintaining multiple alleles at the locus within a population, where one allele is fitter under one condition, and another allele is fitter under a different condition. Signatures of long-term balancing selection are likely to be detected in native populations because of the seasonally fluctuating environments *C. ciliata* is facing [13,44]. In accordance with this theory, the highest genetic diversity of variant SNPs was observed in the native American *C. ciliata* populations (Table S13). Balancing selection is common in nature; however, research on its role in maintaining the crucial standing variations that natural selection could act on, particularly in populations experiencing rapid environmental changes during invasion, is scarce.

In this study, we were particularly interested in the long-term balancing selection acting on the whole genome in the native ranges [13]; however, the balancing selection on a few specific loci could also allow a species to overcome bottleneck during invasion [45]. Widespread balancing selection signatures were identified along the seven chromosomes in the native ranges (Figure 6A). Moreover, candidate SNPs under selection and associated with invasion showed significantly higher *β* scores of long-term balancing selection compared with other genomic regions (Figure 6A; Table S20). This finding further supported the hypothesis that these loci under balancing selection in native ranges may play pivotal roles in establishing *C. ciliata* after introduction into the invasive ranges. The genes under long-term balancing selection overlapped substantially with expanded genes and genes under selection and associated with invasion (Figure 6B). Despite the highly polygenic mechanism of genetic redundancy-mediated thermal adaptation, these genes exhibited specific enrichment in oxidoreductase and metabolism-related functions (Figure 6C; Table S22) [46]. This result further supports the concept that the pre-introduction adaptation of this species originated from long-term balancing selection in its native ranges, potentially contributing to its oxidative stress tolerance, thermal tolerance, and capacity to invade and establish in new habitats.

Notably, several genes and loci were identified as potential targets of selection; however, direct validation of their functions is complicated by the lack of gene silencing and knockout systems in *C. ciliata* and the complexity of such experiments in this small host-specific herbivore. Nevertheless, our study may benefit developmental biology research as it presents the first high-quality Tingidae genome resource. Future studies will provide a more optimized solution for validating the genomic mechanism of adaptation in this species.

## Conclusion

In this study, we assembled the first Tingidae genome, presenting a chromosome-level genome of the sycamore lace bug *C. ciliata*. High-coverage PacBio, Illumina, and Hi-C sequencing were used. We investigated species-specific gene family expansions and contractions, and conducted in-depth resequencing of 402 samples covering native and invasive ranges across three continents. Two independent invasion routes of this lace bug were identified, both of which originated from the USA and subsequently spread through Europe or Japan, forming a contact zone in East Asia. Genomic signatures of selection associated with invasion and long-term balancing selection were identified in the native ranges. These genomic signatures overlapped with each other and with the expanded genes in the *C. ciliata* genome, which were associated with oxidoreductase and metabolic activities. This finding suggests improved oxidative stress and thermal tolerance in *C. ciliata*. This study elucidates the genomic architecture of the extraordinary invasive capacity of *C. ciliata*, providing insights into the mechanisms underlying the successful expansion of invasive species in rapidly changing environments.

## Materials and methods

### DNA extraction and whole-genome sequencing

*C. ciliata* samples were obtained from China Agricultural University, Western Campus (40.0°N, 116.3°E). Only female samples were used for genome sequencing to lower the effects of heterozygosity and sequencing coverage on the quality of genome assembly because *C. ciliata* possesses an XX/XY sex-determination system [20]. DNA extraction was performed using the DNeasy Blood & Tissue Kit (Catalog No. 69504, QIAGEN, Hilden, Germany), and 26.7 μg of high-molecular-weight (HMW) DNA was obtained from 57 female samples. For short-read sequencing, a 350-bp insert size library was constructed and sequenced on the NovaSeq 6000 platform (Illumina, San Diego, CA) in 150 bp paired-end (PE) mode. For continuous long-read (CLR) sequencing, the 20-kb insert size SMRTbell library was constructed and sequenced on the Sequel II platform (PacBio, Menlo Park, CA) at Berry Genomics (Beijing, China).

The Hi-C sequencing library was subsequently prepared. For crosslinking, 108 female samples were fixed with 2% formaldehyde, and the crosslinked DNA was digested using *Mbo*I restriction endonuclease (Catalog No. ER0811, Thermo Fisher Scientific, Wilmington, DE). A standard Illumina library preparation protocol was performed with the ligated DNA sheared to 300–600 bp. The Hi-C library was also sequenced on the Illumina NovaSeq 6000 platform in 150 bp PE mode.

For full-length transcriptome sequencing, 130 female samples were used. TRNzol Universal Reagent (Catalog No. 4992730, TIANGEN, Beijing, China) was used to extract total RNA, and 3.42 μg RNA was obtained, followed by constructing a 1–10 kb + 4–10 kb SMRTbell library. Circular consensus sequencing was performed on the PacBio Sequel II platform. Raw sequencing data were filtered using the standard IsoSeq3 protocol (https://github.com/ylipacbio/IsoSeq3).

### Chromosome-level genome assembly

Illumina sequencing reads were used to estimate the genome size. JELLYFISH [47] was used to analyze the *k*-mer distribution. GenomeScope v1.0 [48] was used to estimate genome size, heterozygosity, and repetitive sequences based on a *k*-mer distribution (*k* = 19).

For genomic assembly, the PacBio CLR data were corrected and trimmed using Canu v1.8 [49]. Wtdbg2 [50] was then used to assemble the trimmed reads (genome size = 400 Mb). To polish the initial assembly with PacBio long reads and Illumina short reads, Arrow (https://github.com/PacificBiosciences) and Pilon (https://github.com/broadinstitute/pilon) were used, respectively. Redundans v0.13c [51] was used to remove the duplicates, producing an improved assembly.

Juicer [52] and 3D-DNA [53] were used to anchor the initial assembly onto seven chromosomes with the Hi-C reads for chromosome-level scaffolding. The sex chromosome was not specifically identified. BUSCO v3.0.1 was used to assess the completeness of the reference genome based on genes in the Insecta odb10 database [54].

### Genome annotation

RepeatMasker v4.07 (http://www.repeatmasker.org) was used to search for the repetitive sequences and transposable elements (TEs) in the *C. ciliata* genome depending on a search in Repbase, and a *de novo* repeat library was constructed using RepeatModeler v1.0.11 (http://www.repeatmasker.org/RepeatModeler). RepeatProteinMask and TRF were used to search for TE proteins and tandem repeats.

Protein-coding gene structures were predicted using homology-based prediction, *ab initio* prediction, and transcriptome-based prediction. For homology-based prediction, GeMoMa v1.6 [55] was applied to the protein sequences extracted from the published *Halyomorpha halys*, *Diuraphis noxia*, *Oncopeltus fasciatus*, and *Rhodnius prolixus* genomes. For *ab initio* prediction, Augustus v3.3 [56], SNAP [57], GlimmerHMM v3.0.4 [58], and GeneMark-ET v4.21 [59] were used. For transcriptome-based prediction, the full-length transcripts obtained were combined with reassembled transcripts based on available short-read RNA sequencing (RNA-seq) data, which were mapped onto our reference genome. The intact open reading frames were predicted using PASA v2.0.1 [60]. Finally, the annotations from these three strategies were integrated using EvidenceModeler (EVM) v1.1.1 [61].

Functional annotation of protein-coding genes was based on homolog searches and the most optimal matches to five public databases. GO, KEGG, and evolutionary genealogy of genes: Non-supervised Orthologous Groups (eggNOG) were annotated using the eggNOG-mapper v2.1.9 [62]. Swiss-Prot and National Center for Biotechnology Information (NCBI) NR annotations were performed using BLASTp (E-value < 1E−05).

MCScan in JCVI (https://github.com/tanghaibao/jcvi/wiki) was applied to identify collinear blocks within the *C. ciliata* genome using the protein-coding gene sequences.

### Comparative genomic analyses

OrthoFinder v2.5.4 [63] was used to identify orthologous gene families. The protein sequences of 10 other paraneopteran species were downloaded from the GenBank database. *F. occidentalis* was used as the outgroup because Thysanoptera (thrips) is strongly supported as sister to Hemiptera based on phylogenomic analysis [64]. First, alternative splicing for each gene was filtered out, maintaining the longest transcript. We aligned the proteins between *C. ciliata* and ten other paraneopteran species using BLASTp (E-value < 1E−05).

MAFFT v7.313 and its L-INS-i algorithm [65] were used to align the protein sequences of the identified single-copy genes. RAxML v8.0.19 [66] was used to construct the phylogenetic tree with 100 bootstrap replicates. MCMCTree in PAML v4.9 [67] was used to estimate divergence time with a two-node calibration (confidence interval) obtained from the TIMETREE database (https://timetree.org). CAFÉ5 [68] was used to compare the generated gene family clusters. TBtools v1.112 [69] was used to perform GO and KEGG pathway enrichments for gene families exhibiting expansion and contraction in the *C. ciliata* genome.

### Population sampling, resequencing, SNP calling, and mitogenomic assembly

In total, 410 *C. ciliata* specimens were collected from 42 populations (*n* = 42) in 10 countries on three continents: China (*n* = 19), South Korea (*n* = 5), and Japan (*n* = 4) in East Asia; the United States (*n* = 4) in North America; and Italy (*n* = 2), Germany (*n* = 2), Switzerland (*n* = 2), France (*n* = 2), Austria (*n* = 1), and Belgium (*n* = 1) in Europe. These populations cover the invasive and native regions of the sycamore lace bug. All samples were collected from the sycamore leaves and stored in absolute ethanol in a −80°C freezer. Another 259 specimens from 35 of the 42 populations and an additional set of four novel populations in China, which were not covered above, were only used for mitogenomic assembly. The 259 individuals were pooled with equimolar-quantity genomic DNA from two other species [22]. Genomic DNA was isolated using the DNeasy Blood & Tissue Kit (Catalog No. 69504, QIAGEN), used to construct 350-bp insert size libraries, and was sequenced on the Illumina NovaSeq 6000 platform in 150 bp PE mode to obtain 8 Gb (∼ 20×) sequencing reads.

BWA-MEM v0.7.12-r1039 (https://github.com/lh3/bwa) was used to map the clean reads onto the assembled reference genome. The genome mapping results of each sample were sorted and converted into the BAM format. Duplicate reads were removed using Picard v2.21.6 (https://github.com/broadinstitute/picard). SNPs were called using GATK v4.1.9.0 [70], and all GVCFs were merged for joint genotyping to generate raw SNPs. Only high-quality biallelic SNPs were retained for subsequent analysis, with missing rate < 0.15, MAF > 0.05, and mean depth > 10×.

Clean reads were also mapped onto the published reference mitogenome using Geneious Prime (https://www.geneious.com) to generate mitogenomes of all samples, with mismatch ≤ 2%, gap ≤ 5 bp, overlap ≥ 40 bp, and similarity ≥ 95%.

### Population genetic structure

For the genomic SNPs, KING v2.2.5 [71] was first used to calculate the kinship coefficient between individual pairs and exclude samples that were predicted to be full-siblings of other samples within the same population (under a threshold of 0.177). Genomic SNPs were pruned, and a single SNP was maintained per 3-kb window to generate independent loci to avoid the LD effects on the inference of genetic structure. SNPs associated with the sex chromosome were not excluded in our analyses. ADMIXTURE v1.3.0 [72] was used to infer the population genetic structure with independent SNPs. Hypothetical ancestral cluster *K* (ranging from 2 to 10) was analyzed to determine the optimum number. PCA was performed using the PLINK software. RAxML was used to reconstruct the phylogenetic relationships of all samples based on the maximum-likelihood (ML) method with the GTR + G model. TreeMix 1.13 [73] was used to infer patterns of historical splits and admixture events among different regions through reconstructing the bifurcating ML tree with 100 bootstraps. PLINK was used to generate allele frequency data. The migration edges (*m* = 2) were modeled from 0 until when 99.8% of the variance of relatedness between populations can be explained by the model (Figure S10).

For the mitogenomes, protein-coding genes, tRNA genes, rRNA genes, and the control region were aligned separately using MAFFT. Bayesian analysis of the population structure was performed using BAPS v6.0 [74] under the spatial clustering of groups of individuals. Phylogenetic analysis was performed based on the haplotypes generated by DnaSP v6.0 [75] (haplotype information shown in Table S23). ML analysis was performed using the ultrafast bootstrap approximation approach in IQ-TREE v1.6.5 [76] with 1000 replicates.

### Genetic diversity and demographic history

VCFtools was used to calculate the genome-wide nucleotide diversity (*θ*_π_) of populations from five different regions, and Weir and Cockerham’s mean estimate of *F*_ST_ based on the sliding-windows along the genome was calculated. The observed and expected homozygosity and heterozygosity were calculated for each sampling population using the populations function in Stacks 2.65 [77]. Mitogenomic diversity was calculated using DnaSP. Arlequin v3.5 [78] was used to calculate pairwise *F*_ST_ values to measure mitogenomic differentiation between different regions.

Twenty randomly selected samples representing every population in the five native and invasive regions (Table S19) were used to estimate the changes in effective population size (*Ne*) in the past using SMC++ [79]. The Stairway Plot 2 method was more robust when reconstructing more recent demographic histories. Thus, Stairway Plot 2 [80] was also used to infer the demographic history. EasySFS was used to convert the VCF format to the site frequency spectrum (https://github.com/isaacovercast/easySFS#easysfs). We applied the mutation rate of *Drosophila melanogaster*, 2.8 × 10^−9^ per base pair per generation, and a generation time of 0.2 because five generations per year can be completed in the wild for *C. ciliata* [81]. We calculated the correlation coefficient (*r^2^*) after increasing decay distance and plotted LD decay curves using PopLDdecay [82] to measure the degree of LD among populations in each region based on the biallelic SNPs filtered out without missing data.

### Selection signature detection

Genomic signatures of selection associated with invasion were analyzed based on filtered SNPs using genome-wide distribution of population fixation statistics *F*_ST_ and nucleotide diversity *θ*_π_. Four pairs of invasive and native populations were selected, and VCFtools was used to calculate *F*_ST_ and *θ*_π_ with 10-kb sliding window and 5-kb step size. Negative *F*_ST_ values were considered to be 0. For *θ*_π_, log_2_  *θ*_π-invasive_/*θ*_π-native_ was further calculated. The divergence regions from the intersection of the top 1% *Z*-transformed *F*_ST_ and log_2_  *θ*_π-invasive_/*θ*_π-native_ were regarded as the putatively selection signatures. The same analyses were performed using 20 randomly selected samples from each region to ascertain whether the number of samples biased the results (Table S19).

Balancing selection signatures were scanned in the native populations to test whether SNPs and genes showing selection signatures in the invasive populations or key genes expanded in the *C. ciliata* genome overlapped with signatures of balancing selection within native ranges. SNPs (MAF < 0.15) were filtered out to prevent false positives. Seven chromosomes were analyzed separately using BetaScan [83] to obtain *β* scores along the genome. Statistical analyses were conducted in R to compare the *β* scores of candidate SNPs with the other genomic regions.

### Environmental association analysis

Nineteen bioclimatic variables were used in EAA to investigate the potential effect of candidate SNPs on the environmental adaptation of *C. ciliata*. Raster 3.5.2 and SpatialPoints 1.4.6 packages in R were used to retrieve the corresponding climatic data for 42 sampling localities (Table S1) from the WorldClim database. Nineteen variables were first filtered based on Pearson’s correlation tests using IBM SPSS Statistics 19.0 (Chicago, IL) to avoid the multi-collinearity of highly correlated variables. The highly correlated independent variables (|*r*| ≥ 0.85) were removed, and the following variables were used for this analysis: BIO1, annual mean temperature; BIO2, mean diurnal range; BIO3, isothermality; BIO4, temperature seasonality; BIO5, maximum temperature of the warmest month; BIO6, minimum temperature of the coldest month; BIO8, mean temperature of the wettest quarter; BIO12, annual precipitation; BIO13, precipitation of the wettest month; and BIO14, precipitation of the driest month. The environmental variables of populations were calculated as the absolute difference between the value of each variable and the average across all populations, standardized by the standard deviation. Bayenv2 [84] was used to investigate the correlation between environmental variables with standardized allele frequencies among populations because of its lower false positive rate and decent performance in handling hierarchical population structure.

## Supplementary Material

qzae074_Supplementary_Data

## Data Availability

The raw sequencing data generated in this study have been deposited in the NCBI Sequence Read Archive (BioProject: PRJNA1026752), as well as in the Genome Sequence Archive [85] at the National Genomics Data Center (NGDC), Beijing Institute of Genomics (BIG), Chinese Academy of Sciences (CAS) / China National Center for Bioinformation (CNCB) (BioProject: PRJCA025090), which are publicly accessible at https://ngdc.cncb.ac.cn/gsa. Detailed information is provided in Table S24. The genome assembly is accessible in the NCBI Genome (GenBank: JAWNBC000000000), as well as in the Genome Warehouse [86] at the NGDC, BIG, CAS / CNCB (GWH: GWHESEE00000000), which is publicly accessible at https://ngdc.cncb.ac.cn/gwh. The genome annotation files, mitogenomic dataset, tree files, and datasets for statistical analyses are available on figshare (https://doi.org/10.6084/m9.figshare.24549202.v1). Bioinformatic software and parameters used in this study are summarized in Table S25.

## CRediT author statement

**Zhenyong Du:** Methodology, Formal analysis, Investigation, Resources, Visualization, Writing – original draft, Writing – review & editing. **Xuan Wang:** Formal analysis, Investigation. **Yuange Duan:** Formal analysis, Investigation. **Shanlin Liu:** Investigation, Writing – review & editing. **Li Tian:** Methodology, Writing – review & editing. **Fan Song:** Resources, Validation. **Wanzhi Cai:** Conceptualization, Supervision, Writing – review & editing. **Hu Li:** Conceptualization, Project administration, Funding acquisition, Writing – review & editing. All authors have read and approved the final manuscript.
