# Supplementary material for: Global Invasion History and Genomic Signatures of Adaptation of the Highly Invasive Sycamore Lace Bug
Source: Genomics Proteomics Bioinformatics. 2024 Oct 14;22(6):qzae074. doi: 10.1093/gpbjnl/qzae074 (PMC11993305; doi:10.1093/gpbjnl/qzae074)
Supplement: qzae074_Supplementary_Data [file qzae074_supplementary_data.zip › supplementary material captions.docx]

**Supplementary materials**

**Figure S1 K-mer coverage frequency plot for the *Corythucha ciliata* genome**

Analysis reveals an estimated genome size of 400 Mb, with a heterozygosity rate of 0.96% and 45.6% repetitive sequences.

**Figure S2 Integrated functional annotation of the *Corythucha ciliata* genome using five databases**

A total of 13,235 genes (86.63%) were annotated by at least one database, with specific annotations as follows: NR database (13,200 genes, 86.40%), SwissProt (8871 genes, 58.06%), KEGG (6441 genes, 42.16%), GO (4226 genes, 27.66%), and eggNOG (12,084 genes, 79.09%). GO, Gene Ontology; KEGG, Kyoto Encyclopedia of Genes and Genomes; eggNOG, evolutionary genealogy of genes: Non-supervised Orthologous Groups.

**Figure S3 GO annotation of protein-coding genes in the *Corythucha ciliata* genome**

GO, Gene Ontology.

**Figure S4 Genetic structure of 370 *Corythucha ciliata* samples as revealed by ADMIXTURE analysis using genomic SNPs, for K = 2–10**

The corresponding CV errors for each K value are: CV error for K = 2 is 0.54354, K = 3 is 0.52886, K = 4 is 0.51886, K = 5 is 0.51416, K = 6 (the lowest) is 0.51208, K = 7 is 0.51251, K = 8 is 0.51258, K = 9 is 0.51293, and K = 10 is 0.51229. The lowest CV error at K = 6 suggests it as the most suitable model for the genetic clustering of these samples. SNPs, single nucleotide polymorphisms; CV, cross-validation.

**Figure S5 PCA plots based on genomic SNPs of 370 samples**

Panel A shows PC1 plotted against PC3, whereas Panel B shows PC2 plotted against PC3. PCA, principal component analysis; SNPs, single nucleotide polymorphisms; CN, China; EU, Europe; JP, Japan; KR, South Korea; US, the United States.

**Figure S6 Genetic structure of Chinese and Japanese populations as revealed by ADMIXTURE analysis using genomic SNPs**

SNPs, single nucleotide polymorphisms.

**Figure S7 PCA plots based on genomic SNPs of samples from five different regions**

All panels display PC1 plotted against PC2. PCA, principal component analysis; SNPs, single nucleotide polymorphisms.

**Figure S8 Stairway plots depicting the demographic history of populations from different regions**

Median estimates from 200 bootstraps are shown in red lines, whereas dark gray lines represent the 75% confidence interval and light gray lines represent the 95% confidence interval of the inference. CN, China; EU, Europe; JP, Japan; KR, South Korea; US, the United States.

**Figure S9 Venn diagram illustrating overlapping genes identified in four pairs of invasive and native populations, each comprising 20 samples**

Please refer to Table S19 for the detailed list of samples included in this analysis.

**Figure S10 Testing of migration edges in the TreeMix analysis**

Two migrations (m = 2) are supported, as they account for 99.8% of the variances and yield the highest *Δm* value.

**Table S1 Overview of sample collection and mitochondrial genome diversity in *Corythucha ciliata* populations**

**Table S2 Genome size data for *Corythucha ciliata***

**Table S3 Detailed statistics and characteristics of predicted protein-coding genes in the *Corythucha ciliata* genome**

**Table S4 List of genes exhibiting expansion and contraction in the *Corythucha ciliata* genome**

**Table S5 GO enrichment analysis results for expanded genes in the *Corythucha ciliata* genome**

**Table S6 KEGG pathway enrichment analysis for expanded genes in the *Corythucha ciliata* genome**

**Table S7 GO enrichment analysis results for contracted genes in the *Corythucha ciliata* genome**

**Table S8 KEGG pathway enrichment analysis for contracted genes in the *Corythucha ciliata* genome**

**Table S9 Genome mapping rates, mean sequencing depth, and coverage statistics for 410 *Corythucha ciliata* samples**

**Table S10 Missing rates for SNPs in *Corythucha ciliata* samples**

**Table S11 Kinship coefficient analysis for *Corythucha ciliata* samples**

**Table S12 BAPS results for mitochondrial genomes of *Corythucha ciliata* samples**

**Table S13 Summary of genetic diversity for 42 populations of *Corythucha ciliata*, based on analysis of whole-genome SNPs**

**Table S14 Analysis of population differentiation among *Corythucha ciliata* across different geographical regions**

**Table S15 List of SNPs identified in selection analysis across *Corythucha ciliata* samples**

**Table S16 List of genes identified in selection analyses across *Corythucha ciliata* samples**

**Table S17 Results of environmental association analysis based on 7,872 candidate SNPs and ten bioclimatic variables**

**Table S18 List and annotation of six shared genes exhibiting genomic signatures of selection**

**Table S19 Identified genes exhibiting signatures of selection, based on a random selection of 20 samples from each region**

**Table S20 Statistical outcomes of Mann–Whitney U tests comparing *β* values of candidate SNPs against those of other genomic regions**

**Table S21 List of genes exhibiting top 1% signatures of balancing selection**

**Table S22 GO enrichment analysis results for genes with overlapping evidence of balancing selection and gene expansion**

**Table S23 List of shared haplotypes identified in the mitochondrial genomes of *Corythucha ciliata***

**Table S24 Information on data uploaded to the NCBI and NGDC databases**

**Table S25 Software and parameters used in this study**
