# Supplementary figures and images for: Global Invasion History and Genomic Signatures of Adaptation of the Highly Invasive Sycamore Lace Bug
Source: Genomics Proteomics Bioinformatics. 2024 Oct 14;22(6):qzae074. doi: 10.1093/gpbjnl/qzae074 (PMC11993305; doi:10.1093/gpbjnl/qzae074)

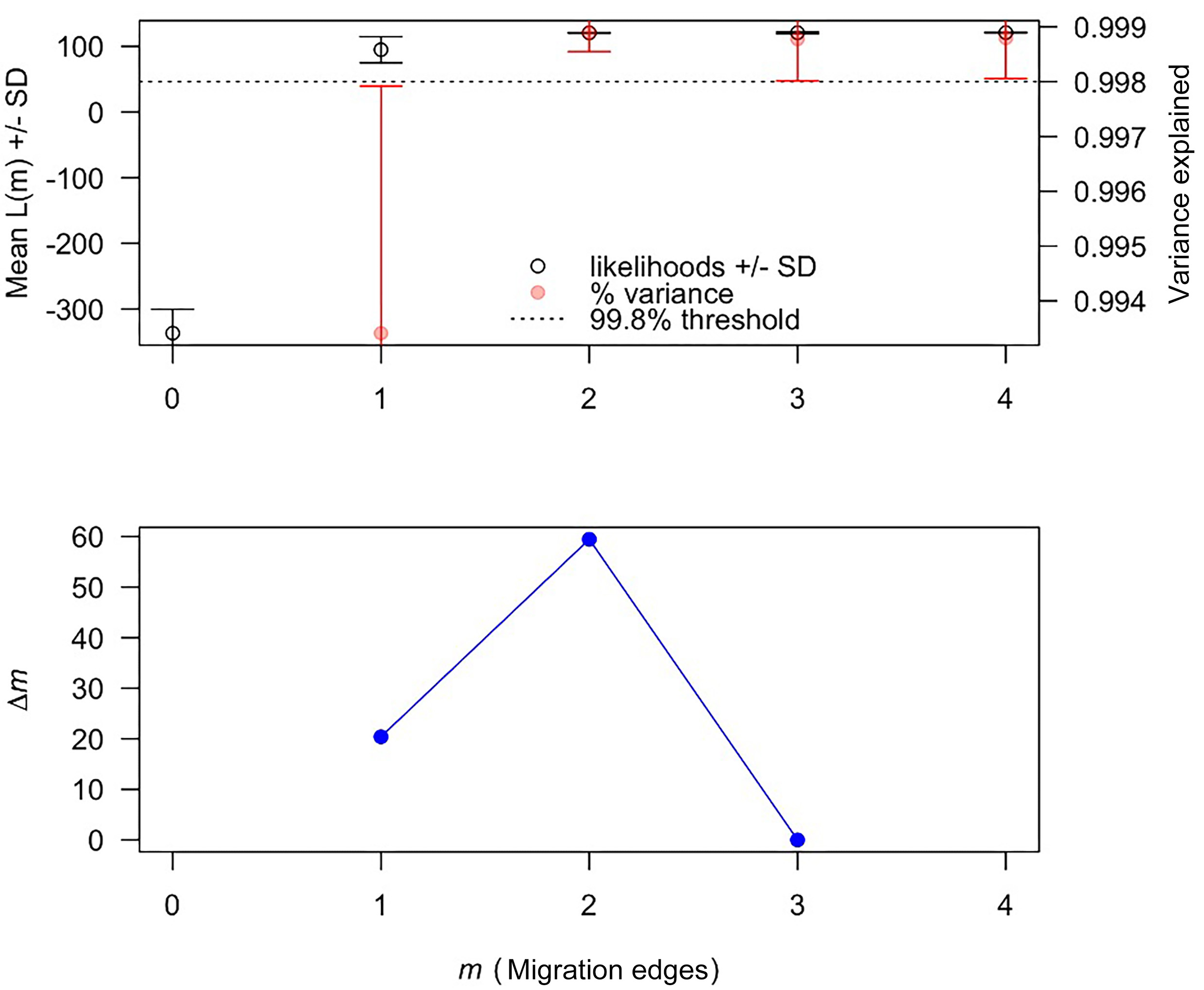

Supplement: qzae074_Supplementary_Data [file qzae074_supplementary_data.zip › Figure S10.jpg]

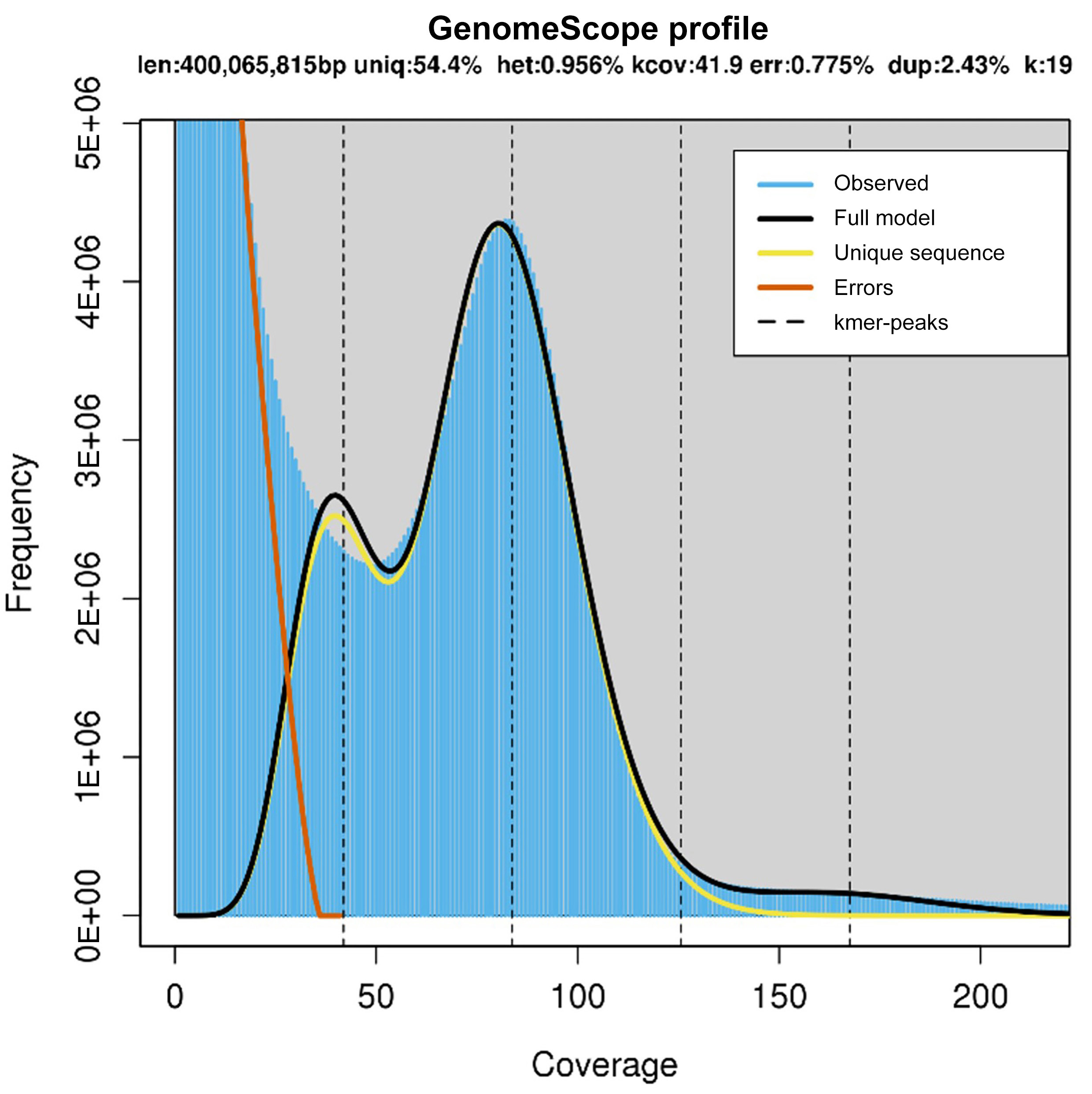

Supplement: qzae074_Supplementary_Data [file qzae074_supplementary_data.zip › Figure S1.jpg]

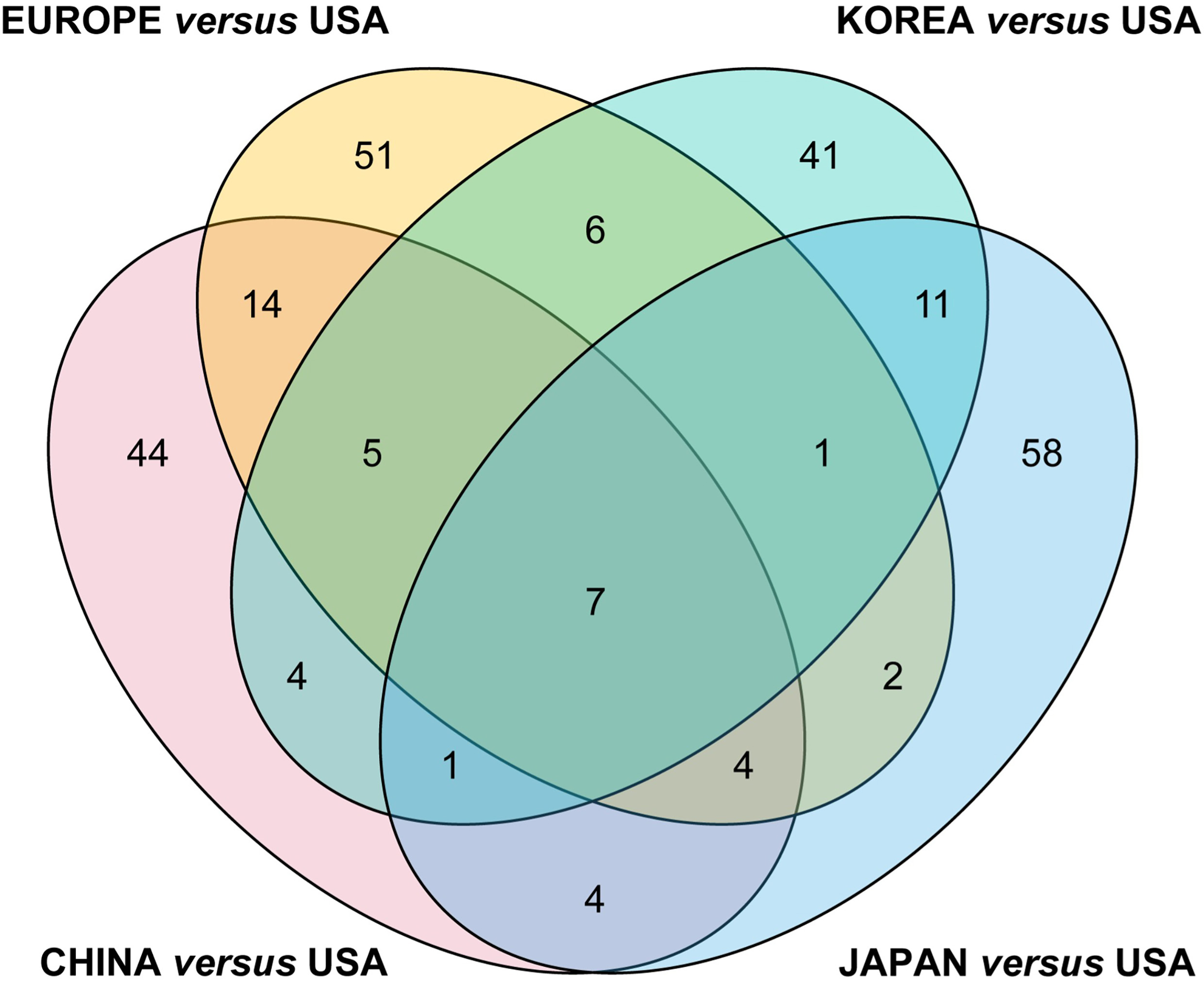

Supplement: qzae074_Supplementary_Data [file qzae074_supplementary_data.zip › Figure S9.jpg]

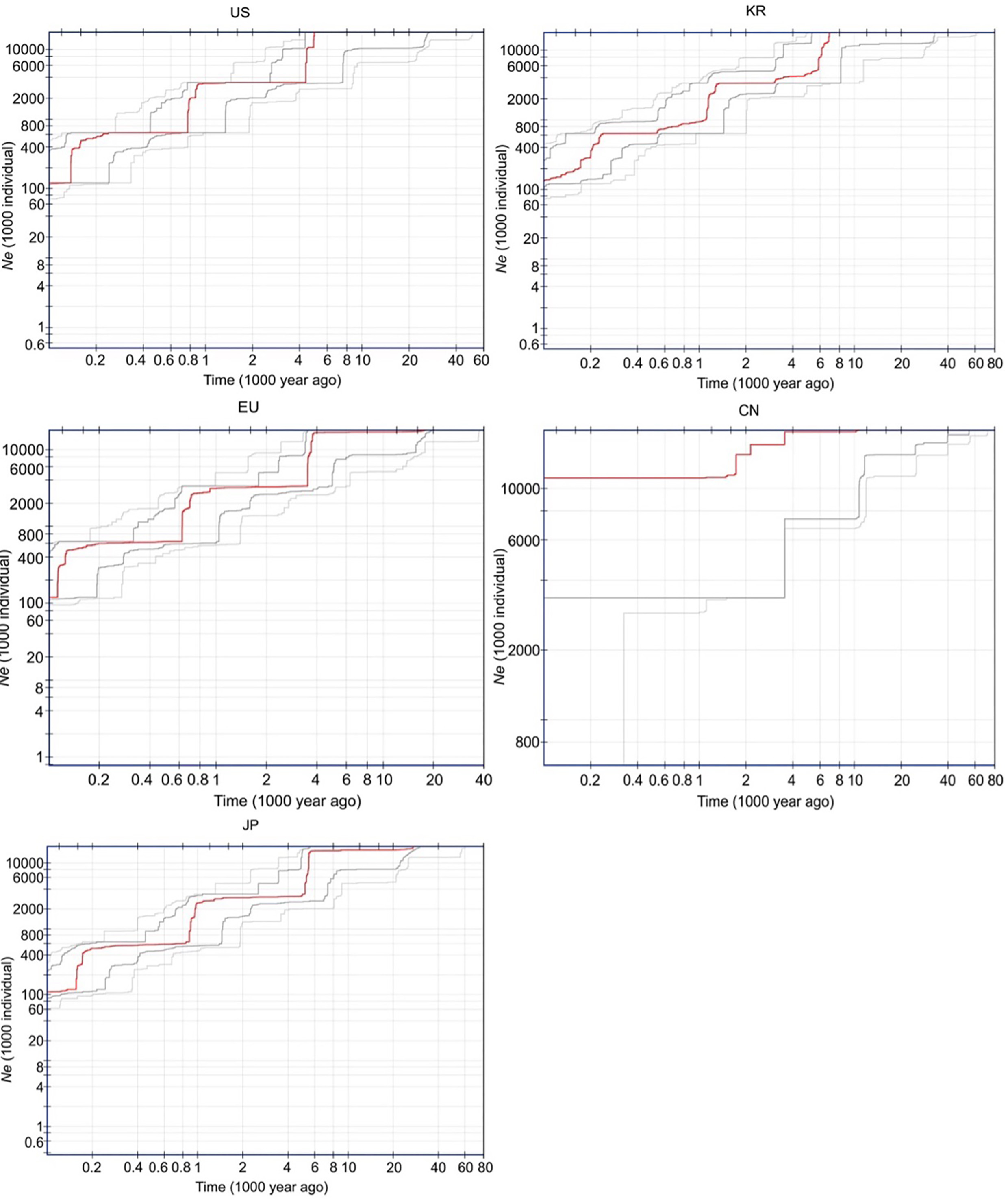

Supplement: qzae074_Supplementary_Data [file qzae074_supplementary_data.zip › Figure S8.jpg]

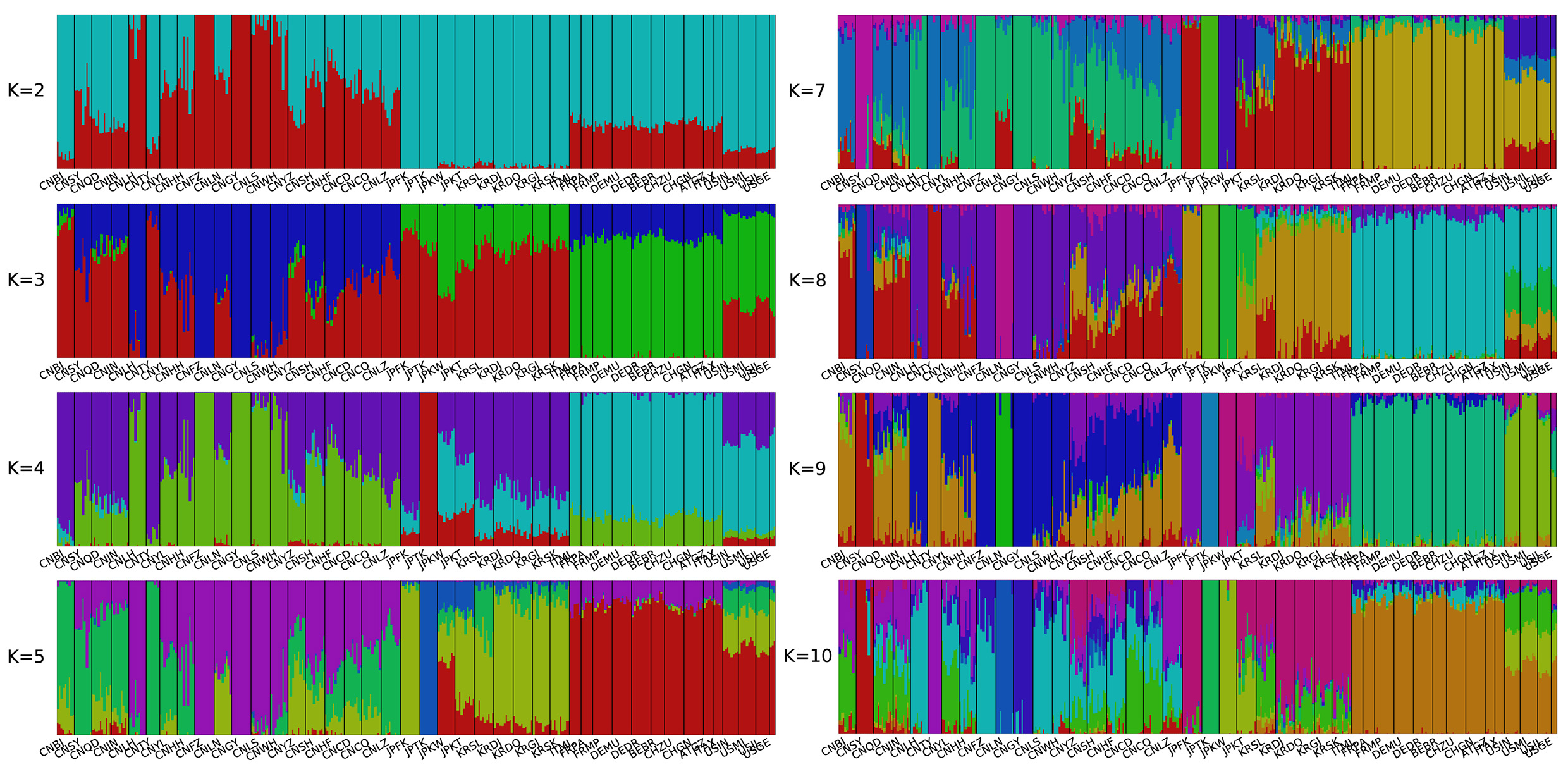

Supplement: qzae074_Supplementary_Data [file qzae074_supplementary_data.zip › Figure S4.jpg]

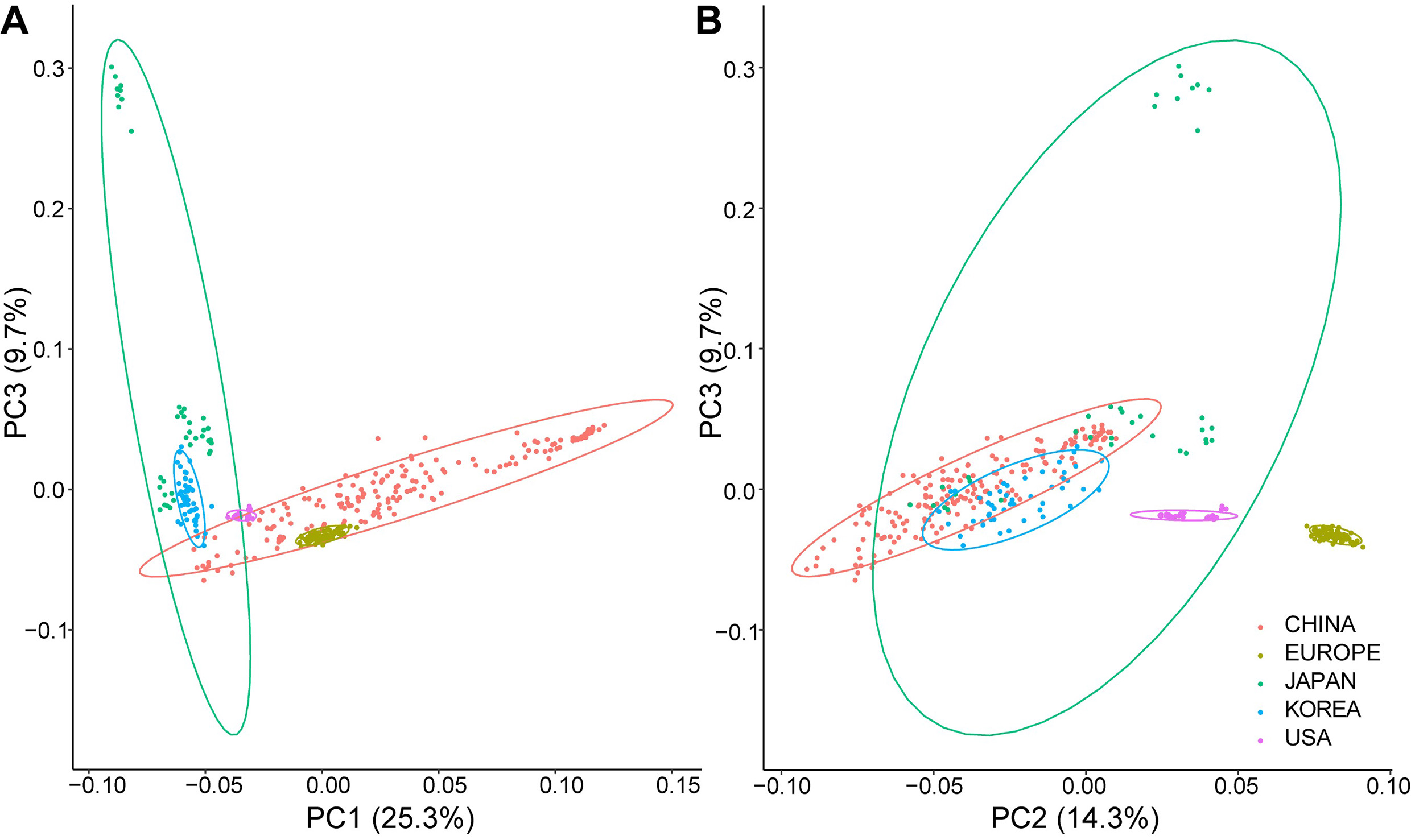

Supplement: qzae074_Supplementary_Data [file qzae074_supplementary_data.zip › Figure S5.jpg]

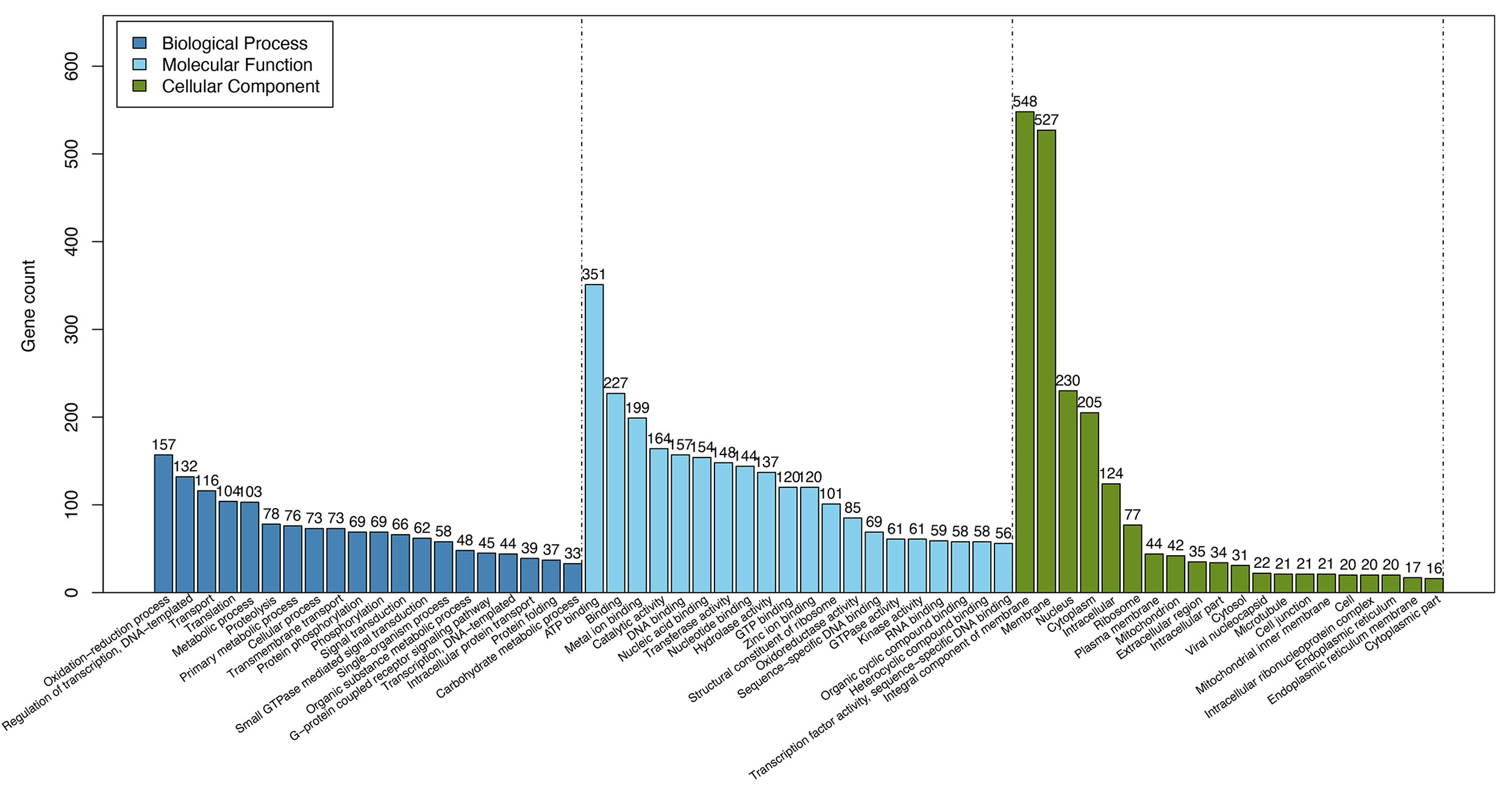

Supplement: qzae074_Supplementary_Data [file qzae074_supplementary_data.zip › Figure S3.jpg]

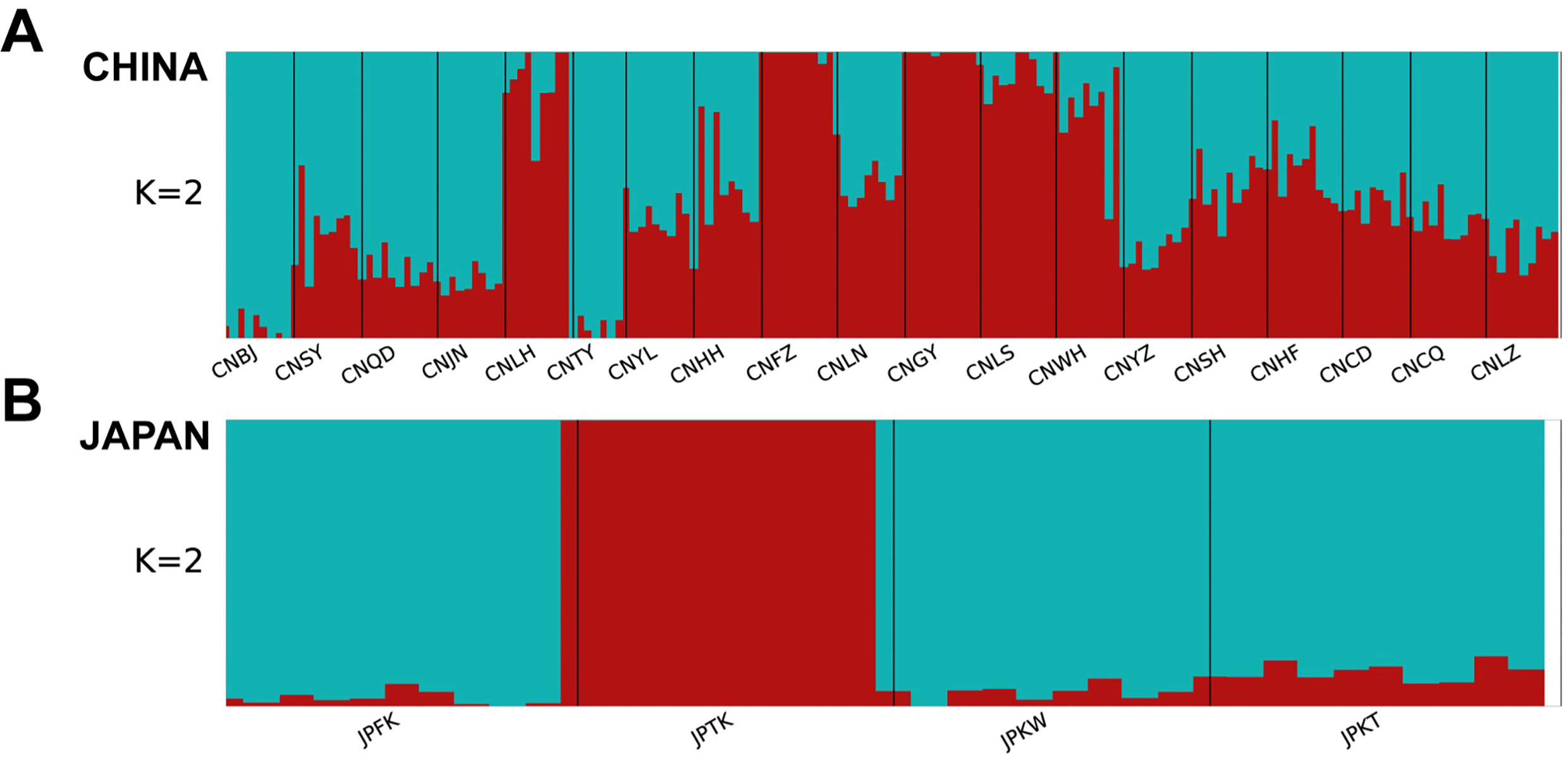

Supplement: qzae074_Supplementary_Data [file qzae074_supplementary_data.zip › Figure S6.jpg]

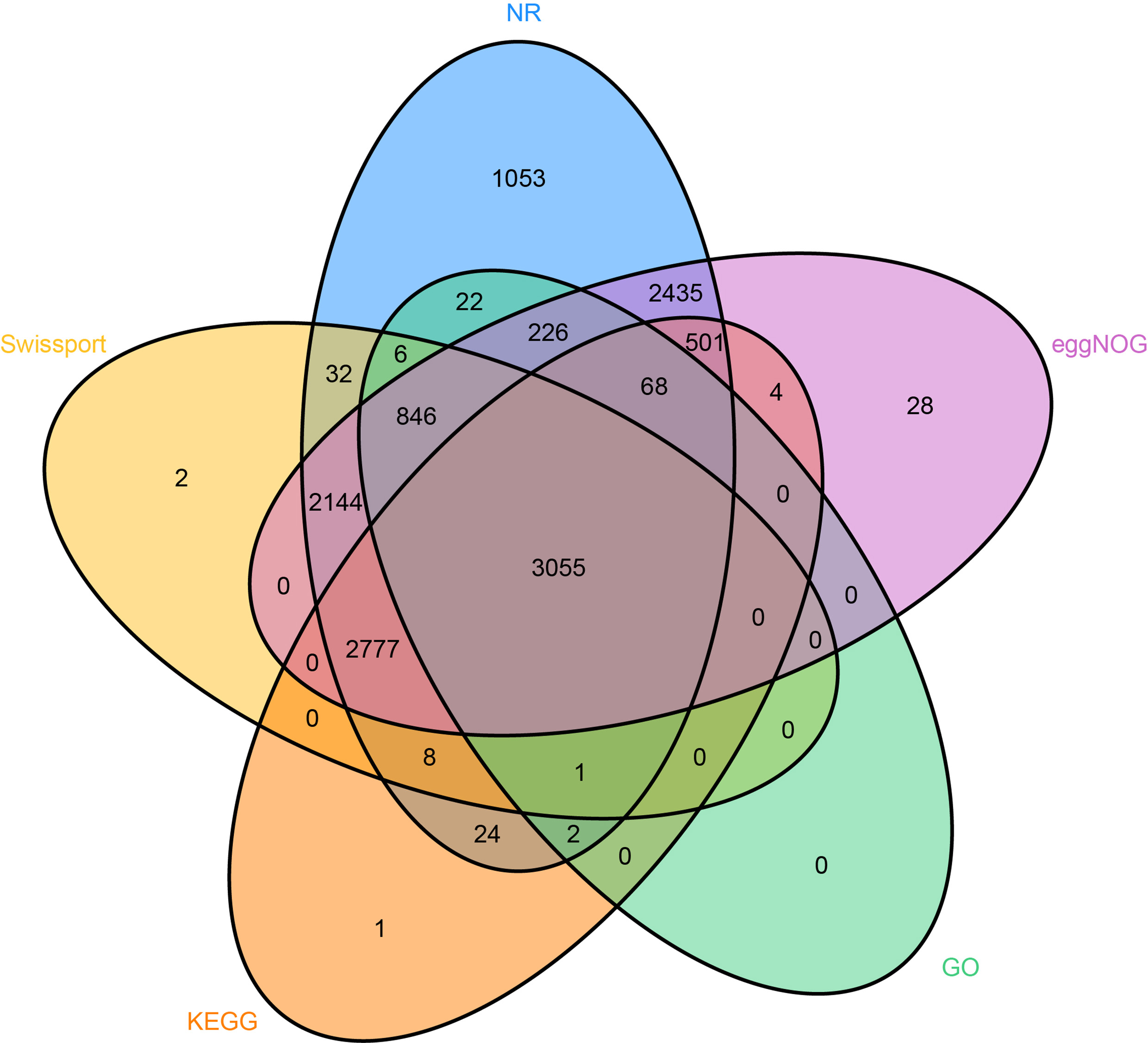

Supplement: qzae074_Supplementary_Data [file qzae074_supplementary_data.zip › Figure S2.jpg]

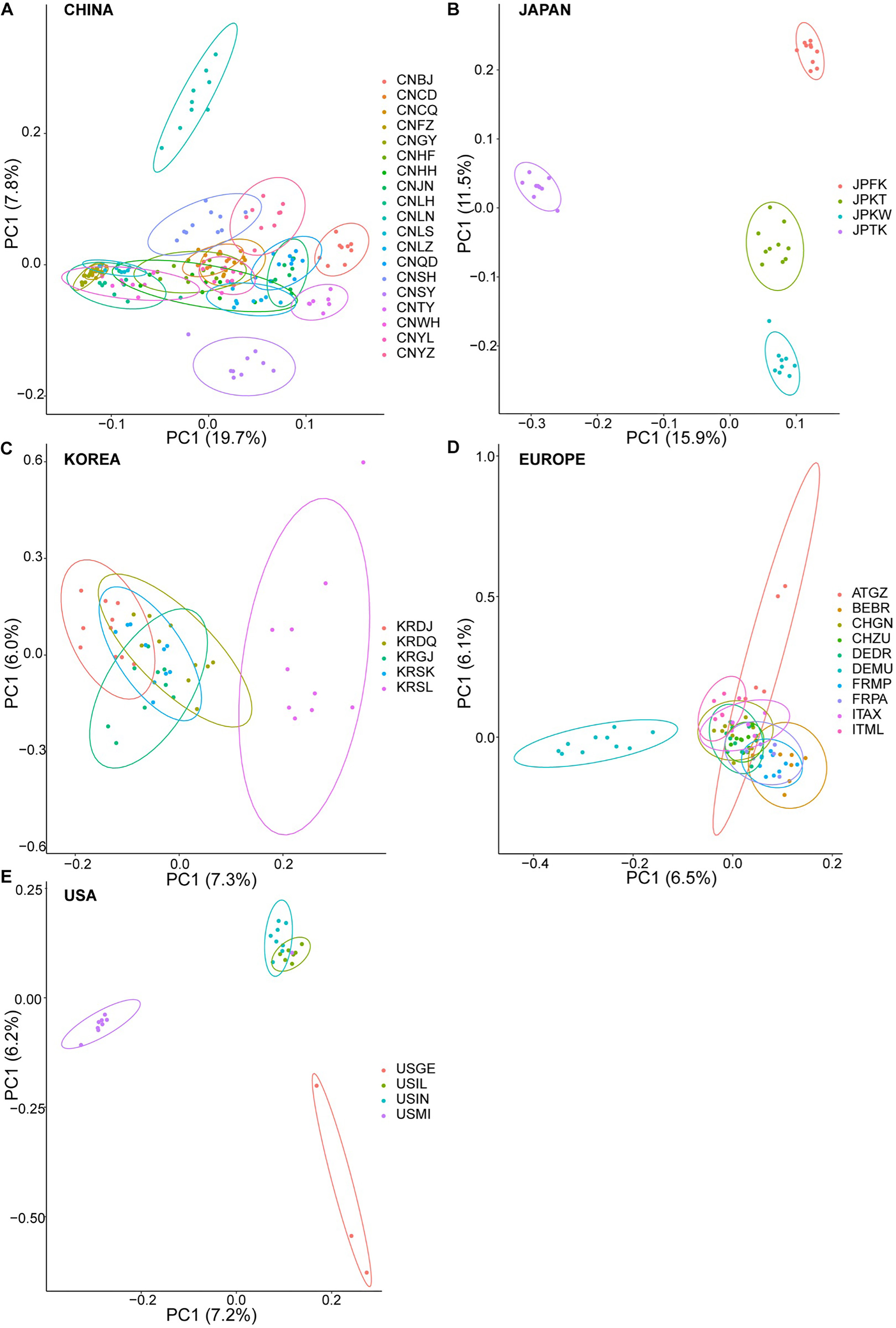

Supplement: qzae074_Supplementary_Data [file qzae074_supplementary_data.zip › Figure S7.jpg]
